# Carboxylate exudation and reproductive effort are associated with leaf phosphorus-resorption efficiency in chickpea

**DOI:** 10.1093/jxb/erag105

**Published:** 2026-02-27

**Authors:** Xiaolong Feng, Huaikang Jing, Chuangwei Fang, Gabriel Crepin, Augustin Dusannier, Jiayin Pang, Peta L Clode, Kadambot H M Siddique, Hans Lambers

**Affiliations:** School of Biological Sciences, The University of Western Australia, Perth, WA 6009, Australia; The UWA Institute of Agriculture, The University of Western Australia, Perth, WA 6009, Australia; School of Biological Sciences, The University of Western Australia, Perth, WA 6009, Australia; The UWA Institute of Agriculture, The University of Western Australia, Perth, WA 6009, Australia; School of Biological Sciences, The University of Western Australia, Perth, WA 6009, Australia; The UWA Institute of Agriculture, The University of Western Australia, Perth, WA 6009, Australia; School of Biological Sciences, The University of Western Australia, Perth, WA 6009, Australia; Institut Polytechnique Unilasalle Beauvais, Beauvais 60000, France; School of Biological Sciences, The University of Western Australia, Perth, WA 6009, Australia; Institut Polytechnique Unilasalle Beauvais, Beauvais 60000, France; School of Biological Sciences, The University of Western Australia, Perth, WA 6009, Australia; The UWA Institute of Agriculture, The University of Western Australia, Perth, WA 6009, Australia; School of Biological Sciences, The University of Western Australia, Perth, WA 6009, Australia; Centre for Microscopy, Characterisation and Analysis, The University of Western Australia, Perth, WA 6009, Australia; The UWA Institute of Agriculture, The University of Western Australia, Perth, WA 6009, Australia; School of Biological Sciences, The University of Western Australia, Perth, WA 6009, Australia; The UWA Institute of Agriculture, The University of Western Australia, Perth, WA 6009, Australia; National Institute of Plant Genome Research, India

**Keywords:** Canopy senescence, P-use efficiency, phenology, phosphorus acquisition, phosphorus resorption, reproductive investment

## Abstract

Efficient leaf phosphorus (P) resorption is a key process that enhances internal P-use efficiency in plants. However, the interactions among leaf P resorption, senescence dynamics, and seed P accumulation remain poorly understood. We evaluated 266 chickpea accessions with diverse genetic backgrounds in a temperature-controlled glasshouse experiment. Measurements included leaf P-resorption efficiency (PRE), reproductive allocation, phenology, canopy senescence traits, and biomass distribution, along with rhizosheath carboxylates, which is an indicator of root P-acquisition strategy. Accessions with higher leaf P concentrations exhibited greater PRE, initiated canopy senescence earlier, and maintained a faster canopy P translocation rate. These combined traits significantly increased aboveground productivity and enhanced P-use efficiency. We also identified a non-linear relationship between carboxylate exudation and PRE: resorption efficiency rose with increased exudation before reaching a plateau. Structural equation modelling revealed that greater carboxylate exudation and reproductive allocation were positively associated with enhanced P resorption. Substantial variation in PRE was observed across accessions, ranging from 70% to 89%. This study is the first to systematically integrate leaf P resorption, root P acquisition, reproductive investment, biomass partitioning, and senescence dynamics. Our findings provide new insights into P-use strategies in chickpea and highlight potential pathways for improving crop P-use efficiency.

## Teaser

Higher leaf-phosphorus chickpea accessions showed greater phosphorus resorption efficiency, earlier senescence, and faster phosphorus translocation, enhancing productivity and phosphorus-use efficiency, while root carboxylate release increased phosphorus resorption efficiency non-linearly.

## Introduction

Phosphorus (P) deficiency is a global challenge limiting primary productivity and crop yield (Veneklaas *et al.*, 2012; Wang *et al.*, 2016). While P fertilization can mitigate this problem, plants typically use only 15–25% of the applied P. The remainder becomes immobilized in soil particles, lost through runoff, or leached into water systems, contributing to soil degradation and eutrophication (Dissanayaka *et al.*, 2018; Paz-Ares *et al.*, 2022). Furthermore, phosphate fertilizers are derived primarily from rock phosphate, a finite resource, making reliance on these inputs both economically and environmentally unsustainable (Fixen, 2009; Sharma *et al.*, 2013). Enhancing crop P-use efficiency (PUE) is therefore critical for sustaining production while reducing environmental impacts.

Phosphorus is initially taken up by roots in the form of inorganic phosphate (Pi) and then is loaded into the xylem via PHO1-family transporters for long-distance transport to the shoot (Fang *et al*., 2024). Within the plant, Pi is subsequently partitioned among tissues and subcellular compartments to support metabolic processes, while excess Pi can be sequestered in vacuoles. Accordingly, diverse phosphate transporters localized in the plasma membrane (e.g. the PHT1 family) and organelle membranes, such as PHT2 in chloroplasts and PHT3 in mitochondria, play essential roles in mediating Pi uptake, intracellular distribution, and remobilization throughout the plant (Yang *et al*., 2024). Plants acquire and use nutrients through both exogenous and endogenous pathways. Exogenous acquisition involves soil uptake via roots or symbiotic interactions with mycorrhizal fungi. Endogenous utilization relies on nutrient recycling from senescing organs, especially leaves, through enzymatic degradation of macromolecules and subsequent redistribution of the released nutrients via the phloem (Smith *et al.*, 2015; Stigter and Plaxton, 2015). This recycling process, particularly P resorption, minimizes nutrient losses before leaf shedding. Because the costs of exogenous and endogenous P acquisition differ, plants adjust their strategies according to resource availability and metabolic ‘economics’ (Bloom *et al.*, 1985; Wright and Westoby, 2003; Raven *et al.*, 2018; Reichert *et al.*, 2022). Species vary in their reliance on exogenous versus endogenous P-use strategies. For example, *Larix principis-rupprechtii* increases root P acquisition under P deficiency (Deng *et al.*, 2016), whereas *Pinus elliottii* relies more on P resorption (Kou *et al.*, 2017). Interestingly, species with higher P-resorption efficiency (PRE) often maintain strong root uptake (de Campos *et al.*, 2013), suggesting that complementary, rather than strict compensatory, strategies are possible. However, crop breeding has historically focused on enhancing root acquisition and grain yield, with limited attention to resorption. A deeper understanding of how P resorption integrates with other traits could enable more systematic improvements in crop PUE.

Senescing leaves are critical sources of P for grain filling (Veneklaas *et al.*, 2012; Smith *et al.*, 2015; Stigter and Plaxton, 2015; Jeong *et al.*, 2017). Studies in wheat, rice, and other cereals have shown that seed P largely derives from remobilization of vegetative stores rather than direct root uptake, even when external P supply is adequate (Julia *et al*., 2016; El Mazlouzi *et al*., 2020, 2022). However, different plant species and even accessions within species vary in the extent to which grain P depends on remobilization versus soil uptake (Girondé *et al.*, 2015; Stigter and Plaxton, 2015). Despite its importance, vegetative-to-reproductive P remobilization has received far less attention than root uptake. Unravelling the mechanisms underlying P resorption will be essential for developing crops with improved PUE and grain yield.

Chickpea (*Cicer arietinum* L.)—the world’s third most widely grown legume globally and the primary cool-season crop cultivated on marginal soils in semi-arid regions—plays a vital role in providing high-quality protein, particularly for vegetarian diets (FAO, 2020). It is extensively grown in regions such as the Mediterranean, Australia, India, and Ethiopia, with its importance poised to increase under changing climatic conditions (Pang *et al.*, 2018a; Varshney *et al.*, 2021). Phosphorus deficiency is a major constraint on chickpea productivity (Srinivasarao *et al.*, 2006), exacerbated by the crop’s symbiotic nitrogen fixation, which increases P demand for optimal nodule function (Meena *et al.*, 2018). Recent research has highlighted chickpea accessions with high PUE, focusing on root architectural traits (Thudi *et al.*, 2021), release of root exudates such as carboxylates (Pang *et al.*, 2018b), and photosynthetic PUE (Pang *et al.*, 2018a; Wen *et al.*, 2020, 2023). However, genotypic variation in PRE—a key internal P-recycling mechanism during leaf senescence—remains largely unexplored. To address this knowledge gap, we examined a diverse chickpea reference set of 266 accessions (260 cultivated and six wild relatives) to characterize variation in leaf P remobilization.

We tested three hypotheses under conditions of mild P deficiency: (i) extended canopy senescence enhances leaf PRE, but reduces canopy P-translocation rate due to limited phloem loading capacity during late senescence; (ii) a trade-off exists between root P acquisition and leaf PRE, where increased P acquisition leads to reduced leaf PRE; and (iii) higher PRE is positively associated with seed yield and seed total P content.

## Materials and methods

### Plant material and growth conditions

This study used a comprehensive set of 266 chickpea accessions, including 203 desi types, 47 kabuli types,10 pea-shaped types, and six wild *Cicer* accessions representing two *Cicer* species (*C. reticulatum* and *C*. *echinospermum*). All plants were grown in 2 litre black pots with four replicates in a temperature-controlled glasshouse at The University of Western Australia, Perth (31°58′ S, 115°49′ E) from April to December 2023 (Supplementary Fig. S1). Average day/night temperatures were 22/15 °C, with a mean relative humidity of 65%. Pots were rearranged periodically to minimize spatial effects.

The growth medium consisted of a 1:4 (w:w) mixture of field soil and sterilized washed river sand. Field soil was collected from the 0–15 cm layer at Cunderdin Agriculture College, 158 km east of Perth. The mixture contained 8 mg kg^−1^ nitrate-N, 1.7 mg kg^−1^ ammonium-N, 10 mg kg^−1^ Colwell P, 6.4 mg sulfur, 135 mg kg^−1^ Colwell K, 9 mg kg^−1^ organic carbon, and had a pH (CaCl_2_) of 7.2. Exogenous P was supplemented at 20 mg kg^−1^ soil as KH_2_PO_4_. Other basal nutrients were applied before sowing, including 15 mg N kg^−1^ soil as Ca(NO_3_)_2_·4H_2_O, 3.75 mg N kg^−1^ soil as NH_4_Cl, 50 mg S kg^−1^ soil as K_2_SO_4_, 0.12 mg B kg^−1^ soil as H_3_BO_3_, 2 mg Zn kg^−1^ soil as ZnSO_4_·7H_2_O, 0.5 mg Cu kg^−1^ soil as CuSO_4_·5H_2_O, 0.4 mg Mo kg^−1^ soil as Na_2_MoO_4_·2H_2_O and 5 mg Fe kg^−1^ soil as FeNaEDTA (Pang *et al*., 2018a; Wen *et al*., 2023). Each pot was filled with 2.5 kg of air-dried soil mixture (see Supplementary Table S1 for more details).

Seeds were coated with fungicide (P Pickel-T®, 360 g l^−1^ thiram, 200 g l^−1^ thiabendazole) before sowing. Four seeds were planted per pot at a depth of 3 cm and later thinned to one plant approximately 15 days after sowing (DAS). A peat-based chickpea inoculant (Group N rhizobium, New Edge Microbials, Albury, NSW, Australia) was applied to planting holes and the soil surface 1 week after sowing. Soil moisture was maintained at 80% of pot capacity by weighing pots every other day. Following pod development, soil moisture was maintained above 70% until harvest. After crop establishment, the soil surface was covered with plastic beads to minimize soil evaporation.

### Biometric analyses

At full expansion of the 11th leaf, both the 10th and 11th leaves were harvested, transported on ice to the laboratory, weighed, and then immediately scanned for leaf area using a flatbed scanner (Epson Perfection v850 pro, Tokyo, Japan) at 300 dpi. Leaf area was analysed with WinRhizo 2009 software (Regent Instruments Inc., Quebec, Canada). Samples were oven-dried at 60 °C for 48 h and analysed for P concentration in green leaves (P_g_).

At the onset of leaf senescence, each pot was enclosed with nylon fruit netting (Perth Megaplas, Perth, Australia) to collect senesced and shed leaves. We also measured the P concentration in leaflets from two senesced leaves corresponding to the position of green leaves. The P concentration in these corresponding leaflets showed a significant positive correlation with that in all senesced leaves (Supplementary Fig. S2). At physiological maturity, plants were cut at the soil surface and separated into leaves, stems, pods, and seeds. Roots were carefully washed. All organs were dried at 60 °C for 96 h and weighed to determine biomass. Senesced leaves were ground, and subsamples were analysed for P concentration (P_s_). Senesced stems were cut into small pieces, thoroughly mixed, and subsampled (P_stem_). All tissues were ground to a fine powder using a GenoGrinder vertical ball mill (Spex SamplePrep, Metuchen, NJ, USA). Approximately 40 mg of each leaf sample was digested (∼40 mg) in HNO_3_–HClO_4_ (3:1 v/v), and P concentration was determined using the malachite green colourimetric method (Motomizu *et al.*, 1983).

The following parameters were calculated:


$$\mathrm{PRE}\;=(P_{g}-P_{s})/P_{s}\times 100\text{\%}$$

$$\mathrm{Remobilized}\;\mathrm{total}\;\mathrm{leaf}\;P\;\mathrm{content}\;=(P_{g}-P_{s})\times \mathrm{leaf}\;\mathrm{DW}$$

Where P_g_ is the P concentration in green leaves, P_s_ is the P concentration in senesced leaves, and DW is the dry weight of all senesced leaves.

PUE was calculated as the ratio of total aboveground biomass to total P content in aboveground organs (leaves, stems, pods, seeds).

Data on rhizosheath carboxylates were obtained from Pang *et al.* (2018b), who assessed 100 chickpea accessions at the seedling stage (7 weeks after sowing). Carboxylate release is a key strategy for P acquisition, and higher levels of carboxylate release can mobilize more soil P, thereby increasing the P concentration in green leaves (see Fig. 4 in Pang *et al*., 2018b; Supplementary Fig. S3). While absolute values of carboxylate release change with plant developement, the relative ranking among accessions is consistent. As shown in Pang *et al*. (2022, Fig. 5B), accessions with high early-stage carboxylate release also maintain higher levels at later stages (reproductive stage). Thus, seedling-stage data are appropriate for representing genotypic differences in this trait.

### Phenological recording

Phenological traits were recorded through regular plant inspections, including vegetative and reproductive milestones and senescence dynamics (Nordt *et al.*, 2021). Time to first flower, 50% flowering, first pod, 50% podding, and maturity were recorded for each accession. Physiological maturity was defined as the stage when all pods turned light yellow. The duration from 50% flowering to maturity was recorded as the reproductive period (Gurumurthy *et al.*, 2022).

### Canopy senescence

Canopy senescence was visually scored weekly using a 0–10 scale, where 0 indicates fully green leaves and 10 indicates complete canopy senescence (100% yellowing). Scoring was performed by a single observer to minimize bias. The extent of leaf yellowing is known to correlate closely with chlorophyll degradation and overall chlorophyll loss, validating the reliability of visual scoring as an indicator of senescence (Luschin-Ebengreuth and Zechmann, 2016; Wu *et al*., 2016). Canopy senescence duration (SD) was defined as the period from the onset of senescence to physiological maturity. A canopy survival curve was fitted to determine canopy half-life (*T*_c50_), and canopy turnover rate was calculated as 1/*T*_c50_ (Suárez, 2010; Gurumurthy *et al.*, 2022). A faster turnover rate reflected shorter canopy longevity. Canopy P-translocation rate was calculated as $(P_{g}-P_{s})/\mathrm{SD}$.

### Statistical analyses

The glasshouse experiment was arranged as a randomized factorial block design (accessions) with four replicates. Trait values were averaged across replicates. Differences in leaf P concentration and PRE among market types and wild accessions were assessed using one-way analysis of variance (ANOVA) with Tukey’s HSD test for post-hoc comparisons. All analyses were performed using the multcomp package, and adjusted *P*-values were obtained via the single-step correction method to control for multiple testing across market types. Due to large phenotypic and phenological differences, only the 260 cultivated accessions were included in subsequent analyses. Canopy senescence dynamics were modelled using a generalized additive model (GAM), with *T*_c50_ defined as the time point corresponding to 50% canopy senescence. The relationship between PRE and rhizosheath carboxylate amount per plant was also modelled using a GAM (R package ‘mgcv’) to account for non-linear trends. Model comparison using AIC indicated that the GAM fit the data better than a linear model.

To evaluate direct and indirect effects of leaf functional traits, reproductive traits, reproductive effort, canopy senescence, and carboxylate exudation on PRE, we used partial least squares structural equation modelling (PLS-SEM; R package ‘plspm’). Missing data were imputed using the missForest algorithm, a non-parametric random forest-based method. The normalized root mean squared error for continuous variables was 0.045, indicating high accuracy (Stekhoven and Bühlmann, 2012). Comparisons between regression models fitted to complete-case and imputed datasets showed minimal differences in coefficients, *P*-values, and adjusted *R*^2^, confirming robustness.

Normality and linearity assumptions were checked before Pearson’s correlation analysis. The Shapiro–Wilk test identified several non-normally distributed variables, which were log-transformed. Linearity was confirmed through scatterplot inspection and regression residuals, and non-linear pairs were further examined using Spearman’s correlation. For models with low explained variance (*R*^2^<0.1) but statistically significant relationships (*P*<0.05), we conducted post-hoc power analyses to assess the sensitivity of the tests. Only models with calculated statistical power greater than 0.9 were reported, ensuring that the reported results are unlikely to be affected by low sensitivity or Type II error. Random forest permutation importance analysis (rfPermute) was then applied to identify predictors significantly associated with PRE, and these variables were retained in the SEM.

Model evaluation was based on 5000 bootstrap resamples. Paths were considered significant if the 95% bootstrap confidence interval excluded zero. Model fit was assessed using the goodness-of-fit index (0.50), which exceeded the commonly accepted threshold of 0.36 (Wetzels *et al.*, 2009). All statistical analyses and visualizations were performed in R version 4.3.0 (R Core Team, 2023).

## Results

### Variation in P resorption

**Fig. 1.**
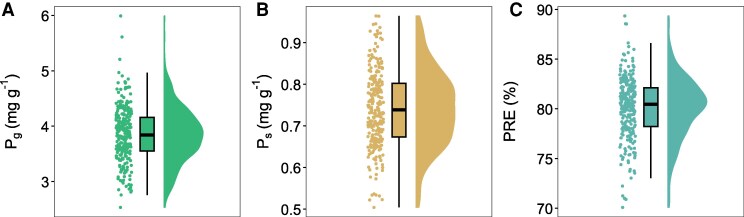
Phosphorus (P) concentration in (A) green leaves (P_g_) and (B) senesced leaves (P_s_), and (C) P-resorption efficiency (PRE) across 260 cultivated chickpea accessions supplied with 20 µg P g^−1^ soil grown in a glasshouse. Raincloud plots show raw data; jittered points (left) and half violins (right) represent individual accession distribution and densities, respectively. Box plots show medians and interquartile range, with whiskers extending 1.5 times the interquartile range.

Substantial variation was observed among the 260 chickpea accessions for all measured traits. P in green leaves (P_g_) ranged from 2.3 to 6.0 mg g^−1^ DW (mean: 3.8), P in senesced leaves (P_s_) from 0.50 to 0.96 mg g^−1^ DW (mean: 0.74), and PRE from 70% to 89% (mean: 80%) (Fig. 1). Aboveground total P content also varied widely, ranging from 16.3 to 49.7 mg—a more than 3-fold difference among accessions. Plant height averaged 61 cm, with a range of 44–85 cm (Table 1). Five accessions (ICC 10775, ICC 1194, ICC 13523, ICC 13219, and ICC 7184) exhibited the highest PRE values (>85%), whereas three (ICC 14799, ICC 16524, and ICC 4393) showed the lowest (<71%).

**Table 1.** Summary of key measured traits and their ranges across 266 chickpea accessions grown in a temperature-controlled glassshouse until physiological maturity

Trait	Mean (range)	Trait	Mean (range)
P_g_ (mg g^−1^)	3.83 (2.33, 5.99)	LMA (g m^−2^)	34.3 (25.3, 66.9)
P_s_ (mg g^−1^)	0.74 (0.50, 0.96)	LDMC (g g^−1^)	0.16 (0.14, 0.26)
PRE (%)	80.0 (70.1, 89.4)	Leaf size (cm^2^)	5.9 (1.6, 10.6)
P_stem_ (mg g^−1^)	0.28 (0.17, 0.75)	DF (DAS)	63 (42, 88)
P_seed_ (mg g^−1^)	3.23 (2.59, 5.18)	DP (DAS)	76 (56, 98)
TP_leaf_ (mg)	3.07 (1.40, 6.46)	DW_leaf_	4.13 (1.86, 6.85)
TP_stem_ (mg)	1.30 (0.55, 3.90)	DW_stem_ (g)	4.67 (2.06, 8.85)
TP_seed_ (mg)	29.6 (13.5, 40.9)	DW_seed_ (g)	9.32 (4.30, 13.34)
TP_aboveground_ (mg)	34.6 (16.3, 49.7)	DW_aboveground_ (g)	20.66 (9.93, 27.66)
PUE	0.60 (0.48, 0.89)	S_start_ (days)	79 (67, 102)
HI	0.45 (0.28, 0.56)	S_end_ (days)	143 (137, 156)
RP	80 (58, 103)	SD (days)	64 (42, 86)
Plant height (cm)	61 (44, 85)	*T*_c50_ (days)	116 (105, 137)

Abbreviations: DAS, days after sowing; DF, days from sowing to 50% flowering; DM days from sowing to maturity; DP, days from sowing to podding; DW, dry weight; HI: harvest index; LDMC, leaf dry matter content; LMA, leaf mass per area; P_g_, P_s_, P_stem_, and P_seed_, phosphorus (P) concentration in green leaves, senesced leaves, stems, and seeds, respectively; RP, reproductive period: days between maturity and flowering; SD, senescence duration; PUE, P-utilization efficiency: the ratio of aboveground DW to aboveground P content; TP_aboveground_, plant aboveground P content, including senesced leaves, senesced stems, pods, and seeds; TP_leaf_, TP_stem_, and TP_seed_, total P content in senesced leaves, senesced stems, and seeds, respectively.

Significant differences in PRE, P_g_, and, P_s_ were detected among chickpea market types. For PRE (*F*_3,260_=4.96, *P*=0.002), the six wild *Cicer* accessions exhibited significantly lower PRE than desi, kabuli, and pea-shaped chickpeas (*P*<0.01), with no significant differences observed among the three cultivated market types (*P*>0.97). Wild species also exhibited significantly lower P_g_ than cultivated accessions (*P*<0.05). Within the cultivated types, kabuli and desi differed marginally in P_g_ but not significantly (*P*=0.066). For Ps, a moderate yet significant market type effect was detected (*F*_3,262_=2.75, *P*=0.043). Kabuli accessions had slightly higher P_s_ than desi (*P*=0.031), while other pairwise comparisons were not significant (Supplementary Fig. S4).

### Correlations among P_g_, P_s_, and PRE

**Fig. 2.**
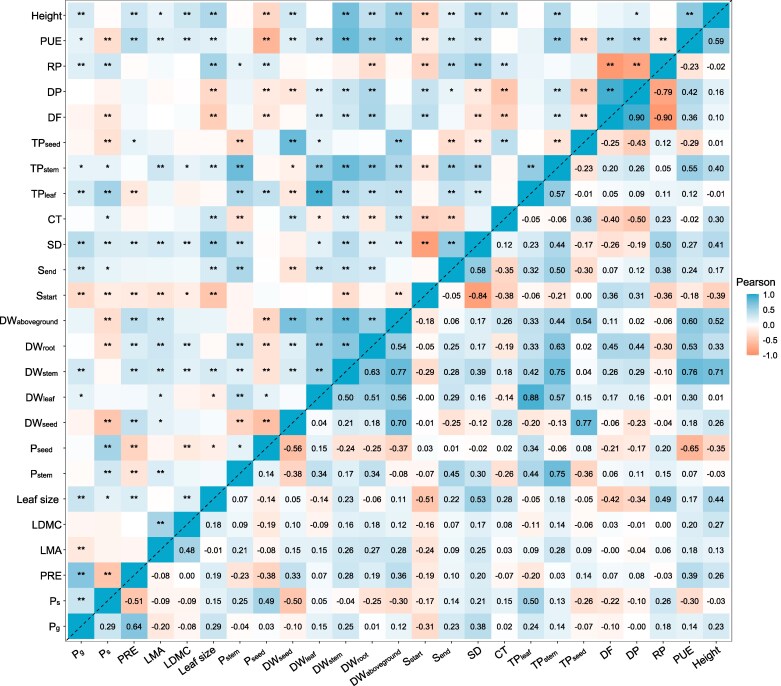
Pairwise Pearson’s correlation among 25 traits measured across 260 chickpea accessions supplied with 20 µg phosphorus (P) g^−1^ soil in a glasshouse. Statistically significant of correlations are indicated as: *P<0.05, **P<0.01. CT, canopy turnover rate; DF, days from sowing to 50% flowering; DM, days from sowing to maturity; DP, days from sowing to podding; DW_aboveground_, aboveground dry weight; DW_leaf_, DW_stem_, DW_root_, DW_seed_, dry weight of leaves, stems, roots, and seed; LDMC, leaf dry matter content; LMA, leaf mass per area; P_g_, P_s_, P_stem_, and P_seed_, P concentration in green leaves, senesced leaves, senesced stems, and seeds; PUE, P-use efficiency; RP, reproductive period: time between DM and DF; S_start_, S_end_, and S_duration_, start, end, and duration of canopy senescence; TP_leaf_, TP_stem_, and TP_seed_, total P content in leaves, stems, and seeds.

PRE positively correlated with P_g_ (*R*^2^=0.41, *P*<0.001) but negatively correlated with P_s_ (*R*^2^=0.27, *P*<0.001) (Supplementary Fig. S5). Additionally, P_s_ was weakly but significantly positively correlated with P_g_ (*R*^2^&0.09, *P*<0.001), with a small effect size (Cohen’s *f*^2^&0.095) (Fig. 2; Supplementary Fig. S6).

### Canopy senescence dynamics and phenology

Canopy senescence followed an S-shaped progression. Approximately 2 months after sowing, the lowest canopy leaves began to senesce while new leaves continued to emerge (Table 1; Supplementary Fig. S7). Once canopy senescence reached ∼10%, the rate of senescence accelerated sharply, coinciding with flowering (*R*^2^&0.10, *P*<0.001) and podding (*R*^2^&0.13, *P*<0.001). Senescence continued for about 2 months until most of the canopy was fully senesced. Senescence duration varied markedly among accessions, ranging from 42 to 86 d, with a mean of 64 d (Table 1; Supplementary Fig. S7).

Substantial variation was also observed in reproductive traits, including flowering and podding dates. Three accessions (ICC 14077, ICC 12654, and ICC 8522) flowered earliest, before 45 DAS, while two accessions (ICC 13077 and ICC 13283) showed delayed flowering, occurring after 85 DAS (Table 1). Podding time strongly correlated with flowering time (*R*^2^&0.81, *P*<0.001), ranging from 56 to 98 DAS across accessions (Table 1; Fig. 2).

**Fig. 3.**
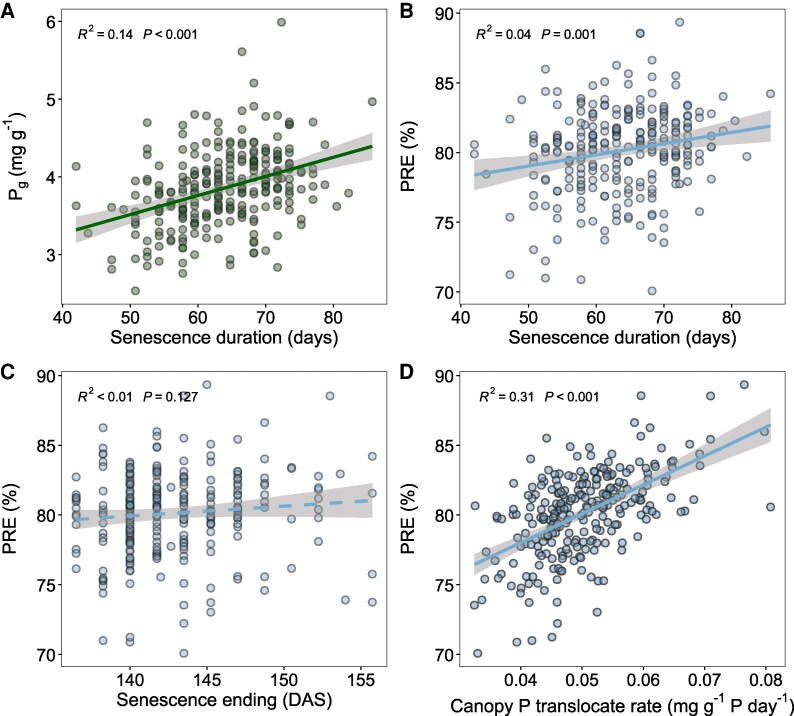
Correlations between green leaf phosphorus (P) concentration (P_g_) and senescence duration (A), phosphorus-resorption efficiency (PRE) and senescence duration (SD) (B), PRE and senescence end date (C), and PRE and canopy canopy P-translocation rate (D) across 260 cultivated chickpea accessions supplied with 20 µg P g^−1^ soil in a glasshouse. Solid lines represent linear regression fits; shaded areas indicate 95% confidence intervals.

Green leaf P concentration negatively correlated with the onset of canopy senescence (*R*^2^=0.10, *P*<0.001), but positively correlated with both senescence end date (*R*^2^=0.05, *f*^2^=0.056, power=0.97, *P*<0.001, Supplementary Fig. S8) and senescence duration (*R*^2^&0.14, *P*<0.001; Fig. 3A). Thus accessions with higher P_g_ tended to canopy senescence earlier but prolonged the process, leading to an extended duration.

PRE positively correlated with senescence duration (*R*^2^&0.04, *P*&0.001, *f*^2^*&*0.043, power&0.91; Fig. 3B), and negatively correlated with the onset of senescence (*R*^2^=0.035, *f*^2^=0.036, power=0.86, *P*=0.003; Fig. 2), but only weakly correlated with senescence end date (*R*^2^<0.01, *P*=0.127; Fig. 3C), suggesting that accessions with higher PRE tended to initiate senescence earlier, but did not significantly delay its completion. Consequently, their longer senescence durations were mainly due to earlier onset rather than later ending. Additionally, PRE positively correlated with canopy P-translocation rate (*R*^2^=0.31, *P*<0.001; Fig. 3D).

### Reproductive efforts and leaf phosphorus resorption

Across the 260 cultivated chickpea accessions, seed P concentration ([P]) varied substantially, ranging from 2.59 to 5.18 mg P g^−1^ DW, with a mean value of 3.23 mg P g^−1^ DW. Eight accessions (ICC 9848, ICC 12379, ICC 11279, ICC 7754, DICC 9100, PBA Slasher, ICC 15248, and ICC 4973) exhibited the lowest seed [P], all below 2.7 mg P g^−1^ DW. In contrast, two cultivated accessions (ICC 440 and ICC 14799) recorded the highest seed [P] among cultivated types (3.84 and 3.78 mg P g^−1^ DW, respectively). Nonetheless, these values were still lower than those observed in five of the six wild accessions, which showed a higher mean seed [P] of 4.2 mg P g^−1^ DW. ICC 7819 and ICC 7554 produced the highest seed yields, exceeding 13 g per plant, whereas ICC 15785, ICC 13077, and ICC 15802 exhibited the lowest yields, each producing less than 6 g per plant.

**Fig. 4.**
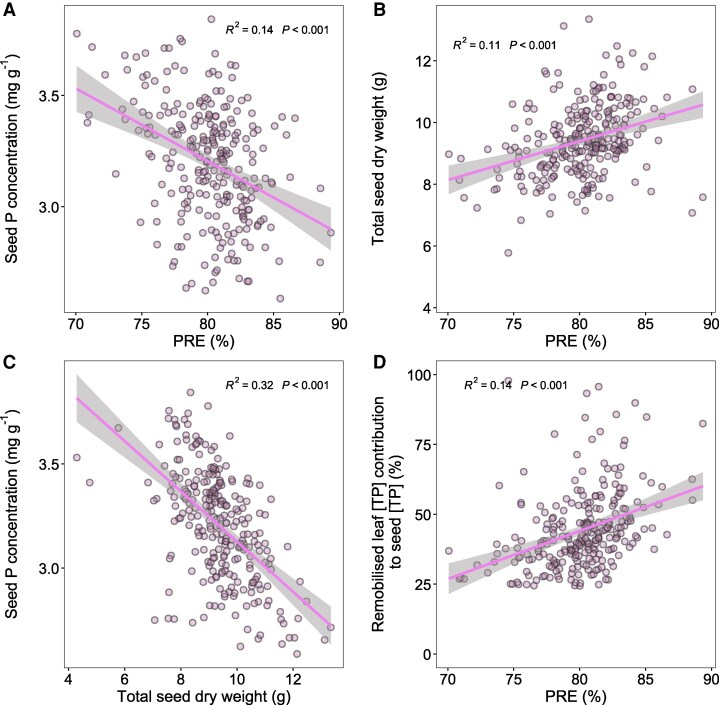
Seed phosphorus (P) concentration (A) and total seed dry weight (B) in relation to phosphorus-resorption efficiency (PRE), seed P concentration in relation to total seed dry weight (C), and the contribution of remobilized total leaf P to total seed P in relation to PRE (D) across 260 cultivated chickpea accessions supplied with 20 µg P g^−1^ soil in a glasshouse. Solid lines represent linear regression fits; shaded areas indicate 95% confidence intervals.

Seed P concentration negatively correlated with PRE (*R*^2^=0.14, *P*<0.001; Fig. 4A) and total seed dry weight (*R*^2^=0.32, *P*<0.001; Fig. 4C). In contrast, total seed dry weight positively correlated with PRE (*R*^2^=0.11, *P*<0.001; Fig. 4B). Importantly, the contribution of P remobilized from senesced leaves to total seed P varied widely among accessions, ranging from approximately 25% to over 75% (*R*^2^=0.14, *P*<0.001; Fig. 4D). This proprtion positively correlated with PRE, indicating that accessions with higher PRE relied more on leaf-derived P to support seed P accumulation.

### Biomass accumulation and phosphorus-use efficiency in relation to leaf phosphorus resorption

Across the 260 chickpea accessions, we detected weak but significant positive correlations between P_g_ and stem dry weight (*R*^2^=0.06, *P*<0.001*, f*^2^=0.067, power=0.99; Supplementary Fig. S9A). Notably, PRE positively correlated with stem dry weight (*R*^2^=0.08, *P*<0.001, *f*^2^=0.084, power>0.99; Supplementary Fig. S9B) and aboveground dry weight (*R*^2^=0.13, *P*<0.001; Supplementary Fig. S9C), suggesting that accessions with higher PRE accumulated more vegetative biomass. Consistent with expectations, PRE positively correlated with PUE (*R*^2^&0.15, *P*<0.001; Fig. 2), demonstrating that enhanced internal P remobilization contributed to both greater biomass production and improved PUE in chickpea.

### Root P acquisition and leaf P resorption

**Fig. 5.**
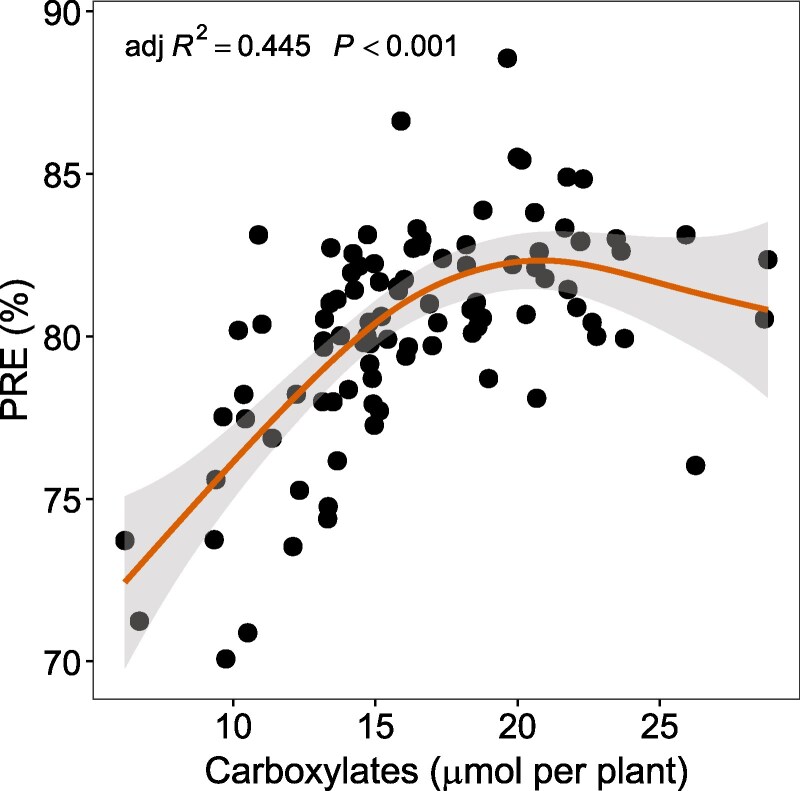
Correlation between phosphorus-resorption efficiency (PRE) and rhizosheath carboxylate release per plant across 98 chickpea accessions (data from Pang *et al*., 2018b). Relationships were modelled using a generalized additive model (GAM). The solid line represents the GAM fit; the shaded area indicates 95% confidence intervals.

We observed a negative correlation between P_s_ and root mass ratio (*R*^2^=0.12, *P*<0.001; Supplementary Fig. S7), and a weak positive correlation between PRE and root dry weight (*R*^2^&0.04, *f*^2^*&*0.039, power&0.89, *P*&0.002; Fig. 2). The GAM further revealed a significant non-linear relationship between PRE and rhizosheath carboxylates per plant (adjusted *R*^2^=0.445, *P*<0.001; Fig. 5; Supplementary Fig. S10). PRE increased with higher rhizosheath carboxylate release up to ∼20 µmol per plant, beyond which the relationship plateaued, indicating a saturating trend (Fig. 5). GAM diagnostics confirmed the adequacy of the model (effective degrees of freedom&2.73, *k*-index&1.07, *P*&0.78), and concurvity values were near zero (<1×10^−20^), indicating no redundancy among terms.

### Correlations among multiple traits

**Fig. 6.**
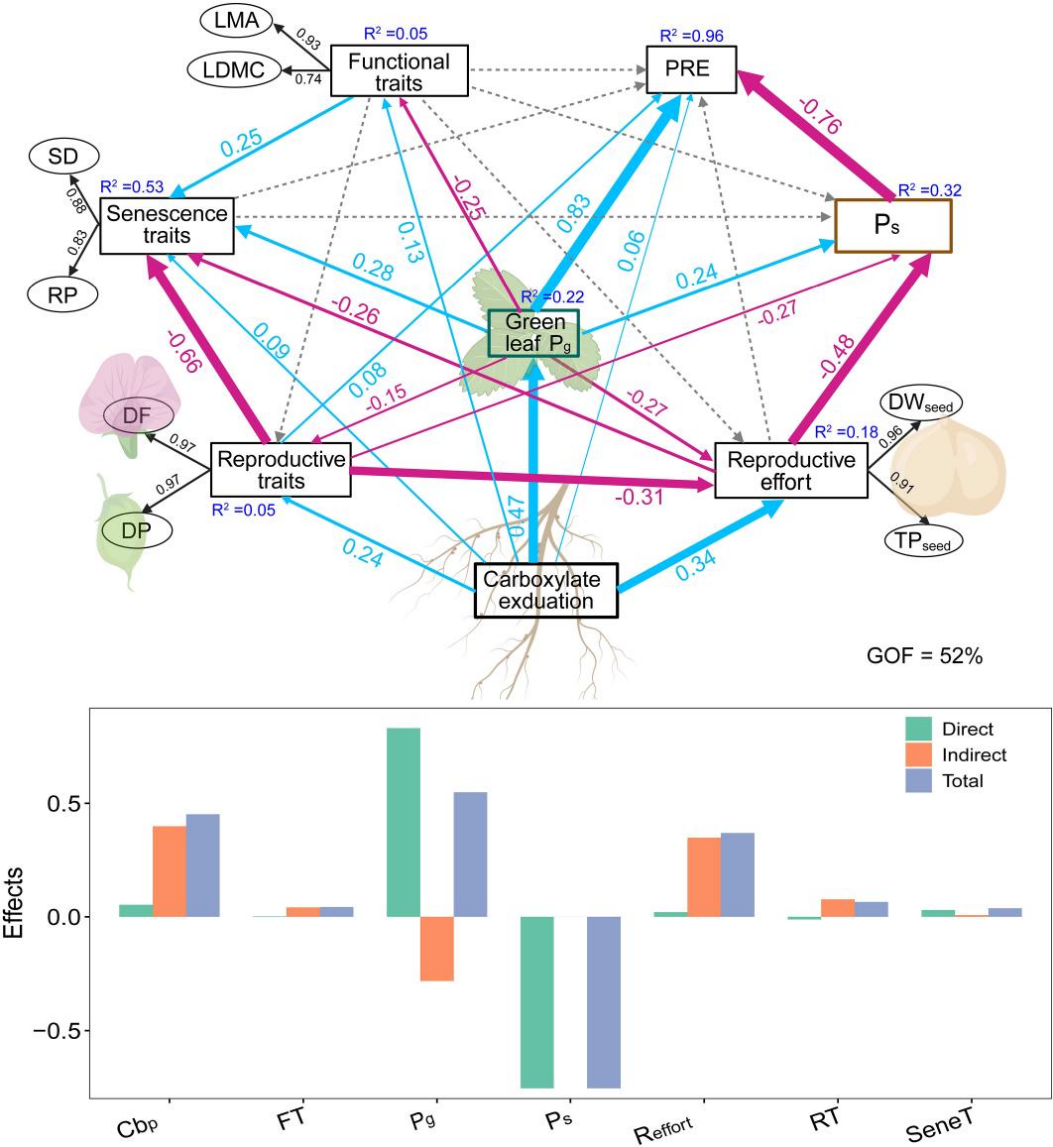
Partial least squares path model (PLS-PM) showing the pathways through which carboxylate amount per plant (Cb_p_), leaf functional traits [FT; leaf mass per area (LMA) and leaf dry matter content (LDMC)], canopy senescence traits [SeneT; canopy senescence duration (SD) and reproductive period (RP)], reproductive traits [RT; flowering date (DF) and podding date (DP)], reproductive effort [REffort; seed dry weight (DW_seed_) and total seed phosphorus (P) content (TP_seed_)], and senesced leaf P (P_s_) contribute to P resorption efficiency (PRE). Arrow width reflects the magnitude of standardized path coefficients (values >0.3 in bold). Blue lines indicate positive effects, red lines indicate negative effects. Solid lines represent significant paths (based on 15 000 bootstraps with 95% confidence intervals excluding zero); dashed lines denote non-significant paths. *R*^2^ values indicate the variance explained for each latent variable. (A) Model fit assessed using the goodness-of-fit (GoF) index; (B) decomposition of total effects of each latent variable on PRE into direct, indirect, and total effects.

Pearson’s correlation analysis revealed significant relationships among leaf functional traits, PUE, canopy senescence dynamics, reproductive traits, reprodctive effort, and biomass allocation (Fig. 2). Leaf mass per area (LMA) positively correlated with leaf dry matter content (LDMC, *r*&0.48, *P*<0.001), but negatively correlated with P_g_ (*r*&−0.20, *P*&0.001). Leaf size positively correlated with P_g_ (*r*&0.29, *P*<0.001), canopy turnover rate (CT, *r*&0.28, *P*<0.001) and PRE (*r*&0.19, *P*&0.002) but negatively correlated with canopy senescence onset (*r*&−0.51, *P*<0.001), time to flowering (DF, *r*&−0.42, *P*<0.001) and time to podding (DP, *r*&−0.34, *P*<0.001; Fig. 6). These findings suggest that accessions with larger leaves tend to have higher P_g_, more efficient P remobilization, and earlier initiation of reproductive development and canopy senescence.

Canopy height positively correlated with stem dry weight (DW_stem_, *r*&0.71, *P*<0.001) and aboveground dry weight (DW_aboveground_, *r*&0.52, *P*<0.001). Canopy turnover rate negatively correlated with DF (*r*&−0.40, *P*<0.001), and DP (*r*&−0.50, *P*<0.001; Fig. 2). PRE negatively correlated with seed P concentration (*R*^2^=0.14, *P*<0.001), but positively (albeit weakly) correlated with total seed P content (*R*^2^=0.02, *f*^2^=0.02, power=0.61, *P*=0.03). In turn, total seed P content strongly correlated with total seed dry weight (*R*^2^=0.59, *P*<0.001) but not with seed P concentration (*P*=0.22, Figs 2, 4).

Partial least squares path modelling (PLS-PM) identified multiple pathways contributing to variation in PRE across cultivated chickpea accessions (Fig. 6). Among all predictors, P_g_ and P_S_ emerged as the strongest direct determinants of PRE (Fig. 6; Supplementary Fig. S11). In addition, rhizosheath carboxylates (a key root P-acquisition trait) and reproductive effort (reflected in total seed dry weight and total seed P content) influenced PRE indirectly by regulating P_g_ and P_S_. Notably, rhizosheath carboxylates also exerted a direct positive effect on PRE, reinforcing their central role in enhancing internal PRE. In contrast, leaf functional traits, canopy senescence dynamics, and reproductive phenology contributed only minor effects in the model (Fig. 6). Collectively, these results suggest that variation in leaf PRE in chickpea is primarily governed by root P-acquisition strategies (carboxylate release) and reproductive P demand.

## Discussion

This study provides the first comprehensive evaluation of how leaf functional traits, canopy senescence dynamics, reproductive traits, reproductive effort, and root P-acquisition strategies are collectively associated with leaf PRE across a large set of chickpea accessions. Most notably, it identified a non-linear relationship between carboxylate exudation—a key P-acquisition strategy—and PRE. This finding challenges the prevailing assumption of either a trade-off or a tight coupling between the two processes (Deng *et al*., 2016; Kou *et al*., 2017; Zhao *et al*., 2020; Wang *et al*., 2025). By integrating both aboveground P allocation and belowground P acquisition, our results provide novel insights into the whole-plant P economy.

### Leaf P concentration and P-resorption efficiency

Green leaf P concentration is widely regarded as a proxy for soil P availability, with higher P_g_ typically reflecting greater soil P supply (Wright and Westoby, 2003). In woody species, higher soil P availability is generally associated with reduced PRE (Kobe *et al.*, 2005; Hayes *et al.*, 2014; Tsujii *et al.*, 2017). In contrast, our results demonstrate a significant positive correlation between P_g_ and PRE in chickpea (Supplementary Fig. S5), suggesting that higher P_g_ did not suppress, but instead enhanced, P resorption—consistent with earlier findings (Killingbeck, 1996; Yang *et al.*, 2020; Chen *et al.*, 2021). The negative correlation between P_s_ and PRE suggests that lower P_s_ values are associated with more complete nutrient resorption from senescing leaves, resulting in higher P-resorption efficiency. This result is consistent with previous studies (Killingbeck, 1996; Kobe *et al*., 2005; Hayes *et al*., 2014).

For most crops, the optimal P_g_ is typically below 4 mg g^−1^ (Veneklaas *et al*., 2012). In our study, the average P_g_ was 3.8 mg g⁻¹, with some accessions reaching up to 6.0 mg g^−1^ (Fig. 1; Table 1). If the additional P was largely stored as inorganic phosphate (Pi) in vacuoles (see Fig. 1 in Veneklaas *et al*., 2012), this pool would represent a metabolically available P that can be readily remobilized and exported via the phloem during leaf senescence. In contrast, organic P forms—such as phospholipids and nucleic acids—require enzymatic hydrolysis by phosphatases before Pi release and phloem transport can occur (Stigter and Plaxton, 2015; Suriyagoda *et al.*, 2023). Future work should examine how leaf phosphorus fractions shift under varying P_g_ levels, with particular attention to the relative contributions of inorganic and organic fractions (e.g., lipid-P and nucleic acid-P) to test this mechanistic explanation.

### Prolonged canopy senescence duration was associated with a high PRE but without compromising the canopy P-translocation rate

During senescence, genes encoding catabolic enzymes are activated, leading to the breakdown of cellular components such as chloroplasts and ribosomes, and the subsequent transport of the degradation products into the phloem (Gregersen *et al.*, 2008; Estiarte *et al.*, 2023). Prolonged senescence can enhance PRE by extending the window for nutrient mobilization. However, this benefit may decline in later stages as energy supply and metabolic activity decrease, potentially slowing P-translocation rate (Stigter and Plaxton, 2015; Veneklaas, 2022).

Our results partially support this hypothesis. We found that prolonged canopy senescence duration was associated with higher PRE, but this did not come at the cost of reduced canopy P-translocation rate (Fig. 3). Although we used green leaves from the vegetative stage to represent initial canopy P status—an approach with some limitations—variation in leaf P concentration during the reproductive stage remains to be further examined. Nevertheless, the strong positive correlation between PRE and canopy P-translocation rate suggests that both processes can be enhanced simultaneously, with no evidence of a trade-off. This finding aligns with Veneklaas (2022), who reported no significant correlation between P resorption and leaf lifespan (or LMA) in the TRY database, implying that species may conserve P through high PRE, extended leaf lifespan, or both strategies.

In this study, accessions with high P_g_ was associated with earlier canopy senescence and exhibited higher PRE. The extended senescence duration was primarily due to earlier onset rather than delayed completion (Fig. 3), suggesting that accessions with high P_g_ and PRE may have extended the senescence window, allowing more time for efficient P recycling from senescing tissues (Stigter and Plaxton, 2015; Veneklaas, 2022). Elevated leaf P concentrations may be associated with earlier senescence by activating senescence-associated genes and P-remobilization pathways, without shortening overall leaf lifespan (Stigter and Plaxton, 2015; Estiarte *et al.*, 2023). Supporting this, Shi *et al.* (2024) showed in *Solanum tuberosum* that the transcription factor StCDF1 regulates the onset of leaf senescence independently of the duration of senescence and total lifespan. Overexpression of *StORE1S02* in a long-lived genotype triggered earlier senescence onset, while overall lifespan remained extended. Thus, early initiation of senescence represents a highly conservative strategy for efficient P conservation, especially under P-deficient conditions.

The strong positive association we observed between PRE and PUE highlights the central role of internal P recycling in supporting chickpea growth under P-limited conditions (Fig. 2). This finding demonstrates that the strategy of efficient P remobilization enhanced aboveground biomass accumulation without relying solely on external P uptake (Killingbeck, 1996; Veneklaas *et al.*, 2012; Gerdol *et al.*, 2019). By reallocating P from senescing tissues to developing organs and seeds (Song *et al.*, 2014; Gao *et al.*, 2017), plants can meet reproductive P demands and improve PUE fitness under nutrient stress. Notably, an increase in PRE was not linked to reductions in canopy turnover rate (Fig. 2), further confirming that higher PRE did not compromise P-translocation capacity (Fig. 3D). Enhanced activities of phosphatases, nucleases, and autophagy-related processes may explain this improved PRE.

We also found that canopy turnover rate positively correlated with plant height and aboveground biomass (Fig. 2), suggesting that faster canopy turnover may support greater aboveground productivity (Veneklaas, 2022). Collectively, these results indicate that selecting for high PRE accessions represents a promising breeding strategy to improve PUE under P-limiting conditions. Rather than focusing solely on improving photosynthetic PUE (Wen *et al.*, 2023), prioritizing internal nutrient recycling through enhanced PRE could deliver greater benefits for sustainable crop productivity (Veneklaas *et al.*, 2012; Wang *et al.*, 2016; Chen and Liao, 2017). Further research should explore whether PUE and PRE are synergistic, independent, or constrained by physiological trade-offs, and examine their underlying genetic linkages.

### Non-linear correlation between P-resorption efficiency and carboxylate exudation

Our results provide the first evidence of a non-linear correlation between a key P-acquisition strategy—carboxylate exudation—and PRE, leading us to reject our second hypothesis. Previous studies have reported diverse correlations between P-acquisition traits and PRE, including trade-offs (Deng *et al.*, 2016; Kou *et al.*, 2017; Zhao *et al.*, 2020), positive associations (de Campos *et al.*, 2013; Lin *et al.*, 2020; Wang *et al.*, 2025), and no consistent pattern (Yang *et al.*, 2025). Similarly, in alpine coniferous forests (Ding *et al*., 2023), PRE has been positively associated with P-mining-related enzyme activity, such as acid phosphatase, and negatively correlated with root uptake traits, such as higher specific root length, specific root area, and mycorrhizal colonization rate. These findings indicate that root nutrient acquisition is influenced not only by morphological traits but also by functional interactions with mycorrhizal fungi, emphasizing the complex trade-offs and synergies within the root economic space (Bergmann *et al.*, 2020). Future studies exploring the link between root P acquisition and leaf nutrient resorption should adopt a holistic approach within this root economic framework. Additionally, investigating the expression of key marker genes related to senescence and carboxylate exudation in selected high- and low-PRE chickpea accessions would provide functional insights into the underlying genetic mechanisms.

In our study, PRE increased with carboxylate exudation per plant, a key P acquisition strategy in chickpea (Pang *et al.*, 2018b), before reaching a plateau (Fig. 5). Additionally, P-resorption proficiency increased with root mass ratio (Supplementary Fig. S12), an important resource-allocation strategy for P uptake (Wen *et al*., 2020). Together, these findings indicate a coordinated relationship between P acquisition and resorption. However, PRE initially increased and then plateaued at high levels of root P acquisition (Fig. 5). We propose three potential explanations for this non-linear correlation. (i) Physiological constraints on maximum PRE: there appears to be an upper threshold beyond which PRE cannot further increase, likely due to essential structural roles of certain P-containing organelles that remain unrecoverable during senescence (Estiarte *et al.*, 2023). For instance, mitochondria must remain functional to supply energy and carbon skeletons until senescence is complete, yet they contain high concentrations of P in their protein-rich matrix and phospholipid membranes (Horvath and Daum, 2013; Chrobok *et al.*, 2016; Estiarte *et al.*, 2023). In our study, PRE substantially exceeded the global average of 65% (Vergutz *et al.*, 2012), and the plateau beyond the inflection point may reflect structural limitations on further increases the PRE. (ii) Minimal nitrogen limitation: hydrolysis of organic compounds to release P requires nitrogen for enzyme synthesis, and root P uptake depends on nitrogen availability for the synthesis of P transporters and associated enzymes (Treseder and Vitousek, 2001). Under nitrogen-limited conditions, plants often face a trade-off between P acquisition and resorption (Wright and Westoby, 2003; Raven *et al.*, 2018). However, chickpea’s inherent nitrogen-fixing capacity allows simultaneous increases in both P-acquisition and P-resorption efficiency, without being constrained by nitrogen availability. (iii) Biomass accumulation and feedback mechanisms: enhanced root P acquisition promotes aboveground biomass and seed yield potential. The resulting increase in vegetative and reproductive sink strength (Tully *et al.*, 2013; Yu *et al.*, 2022) likely drives internal P remobilization to meet the nutrient demands of developing organs. Understanding the mechanisms underlying this coordination between high P-acquisition and P-resorption efficiency provides valuable insights for breeding P-efficient chickpea cultivars. Such cultivars could reduce dependence on P fertilizers and associated environmental impacts while maintaining high yields.

While our root carboxylate data were obtained at the seedling stage, we consider them relevant to PRE for several reasons. First, leaf sampling occurred at an early developmental phase, during which seedling carboxylate exudation is expected to enhance root P acquisition and, in turn, green leaf P concentration—the key indirect pathway linking carboxylates to PRE supported by our SEM (Fig. 6). Second, independent studies show that rankings of accessions for carboxylate exudation are generally consistent across early growth stages and in different environments (Wen *et al*., 2020; Pang *et al*., 2022). Nevertheless, profiling carboxylate dynamics across more accessions and developmental stages would further strengthen the generality of this association. Future work examining the ontogenetic trajectory of root exudation and remobilization will help clarify these interactions.

### Phosphorus resorption and reproductive efforts

Our results support the third hypothesis: higher PRE is associated with increased seed yield and seed total P content. This finding is consistent with previous studies. For example, the P transporter GmPT1, expressed in both leaves and roots of soybean, positively correlated with seed yield, P-acquisition efficiency (shoot P content) and PUE, indicating that enhanced P translocation supports higher seed production (Song *et al.*, 2014). Similarly, female *Populus euphratica* trees exhibit higher PRE than male trees (Yu *et al.*, 2022). In line with this, trees with greater reproductive demand tend to show higher P-resorption proficiency (Tully *et al.*, 2013), suggesting that strong reproductive demand drives more efficient P remobilization from senescing organs.

We also observed a significant negative correlation between seed P concentration and seed dry weight (Fig. 4C), implying that increased seed mass dilutes P concentration. This finding also indicates that chickpea accessions prioritize producing more seeds rather than allocating more P to individual seeds when total P acquisition is higher. Notably, accessions with higher PRE exhibited a greater proportion of seed P derived from senesced leaves, highlighting that seed P in these accessions relied heavily on remobilization from vegetative organs. We propose that remobilized P is first used to support vegetative growth and overall productivity. As reproductive demand increases, P and carbon stored in vegetative tissues are translocated efficiently to developing seeds. This coordinated strategy supports plant vigour and higher seed production under limited external P inputs, emphasizing the adaptive value of efficient internal P recycling.

## Conclusion

Chickpea accessions with higher green leaf P concentration exhibited higher PRE, initiated canopy senescence earlier, and simultaneously maintained a more rapid canopy P-translocation rate. Together, these traits contributed to greater PUE. Notably, this study is the first to reveal a non-linear correlation between a key P-acquisition strategy—carboxylate exudation—and leaf PRE, challenging the conventional view of a strict trade-off or tight coupling between the two processes. We further show that enhanced carboxylate exudation for P acquisition, combined with greater reproductive investment, promotes efficient leaf P resorption, despite substantial variation in PRE among accessions (70–89%). These values are considerably higher than those reported for most other crops and global plant averages. Overall, our findings advance understanding of the plant P economy—including both acquisition and utilization strategies—and provide a framework for breeding programmes aiming to improve internal PUE while maintaining or increasing crop yield.

## Supplementary Material

erag105_Supplementary_Data

## Data Availability

All data that support the findings of this study are available at the public repository: https://github.com/Franke-ovo/Accessions .

## Supplementary data

The following supplementary data are available at *JXB* online.

Fig. S1. Photographs of experimental plants cultivated under controlled greenhouse conditions.

Fig. S2. Correlation between the phosphorus concentration of all senesced leaves and senesced leaflets at the corresponding green leaf position.

Fig. S3. Correlation between green leaf phosphorus concentration and rhizosheath carboxylate release per plant.

Fig. S4. Box plots showing green leaf phosphorus concentration, senesced leaf phosphorus concentration, and phosphorus-resorption efficiency.

Fig. S5. Correlations between phosphorus-resorption efficiency and phosphorus concentration in green and senesced leaves.

Fig. S6. Pairwise Spearman correlation among 25 traits measured.

Fig. S7. Changes in the proportion of canopy senescence during plant development.

Fig. S8. Canopy senescence onset, completion, and duration in relation to green leaf phosphorus concentration.

Fig. S9. Correlations between green leaf phosphorus concentration and stem dry weight and between phosphorus-resorption efficiency and total stem and aboveground dry weight.

Fig. S10. Correlation between phosphorus-resorption efficiency and rhizosheath carboxylate release per plant under original and simulated conditions.

Fig. S11. Results of the random forest model predicting phosphorus-resorption efficiency.

Fig. S12. Correlations between senesced leaf phosphorus concentration and root mass ratio.

Table S1. Composition of basal nutrients applied before planting.

## References

[erag105-B1] Bergmann J, Weigelt A, van der Plas F, et al 2020. The fungal collaboration gradient dominates the root economics space in plants. Science Advances 6, eaba3756. 32937432 10.1126/sciadv.aba3756 PMC7458448

[erag105-B2] Bloom AJ, Chapin FS, Mooney H A. 1985. Resource limitation in plants—an economic analogy. Annual Review of Ecology and Systematics 16, 363–392.

[erag105-B3] Chen H, Reed SC, Lü X, Xiao K, Wang K, Li D. 2021. Coexistence of multiple leaf nutrient resorption strategies in a single ecosystem. The Science of the Total Environment 772, 144951. 33571760 10.1016/j.scitotenv.2021.144951

[erag105-B4] Chen L, Liao H. 2017. Engineering crop nutrient efficiency for sustainable agriculture. Journal of Integrative Plant Biology 59, 710–735. 28600834 10.1111/jipb.12559

[erag105-B5] Chrobok D, Law SR, Brouwer B, et al 2016. Dissecting the metabolic role of mitochondria during developmental leaf senescence. Plant Physiology 172, 2132–2153. 27744300 10.1104/pp.16.01463 PMC5129728

[erag105-B6] de Campos MCR, Pearse SJ, Oliveira RS, Lambers H. 2013. Downregulation of net phosphorus-uptake capacity is inversely related to leaf phosphorus-resorption proficiency in four species from a phosphorus-impoverished environment. Annals of Botany 111, 445–454. 23293017 10.1093/aob/mcs299 PMC3579450

[erag105-B7] Deng M, Liu L, Sun Z, Piao S, Ma Y, Chen Y, Wang J, Qiao C, Wang X, Li P. 2016. Increased phosphate uptake but not resorption alleviates phosphorus deficiency induced by nitrogen deposition in temperate *Larix principis-rupprechtii* plantations. New Phytologist 212, 1019–1029. 27400237 10.1111/nph.14083

[erag105-B8] Ding J, Ge W, Liu Q, Wang Q, Kong D, Yin H. 2023. Temperature drives the coordination between above-ground nutrient conservation and below-ground nutrient acquisition in alpine coniferous forests. Functional Ecology 37, 1674–1687.

[erag105-B9] Dissanayaka DMSB, Plaxton WC, Lambers H, Siebers M, Marambe B, Wasaki J. 2018. Molecular mechanisms underpinning phosphorus-use efficiency in rice. Plant, Cell & Environment 41, 1483–1496. 10.1111/pce.13191 29520969

[erag105-B10] El Mazlouzi M, Morel C, Robert T, Chesseron C, Salon C, Cornu J-Y, Mollier A. 2022. The dynamics of phosphorus uptake and remobilization during the grain development period in durum wheat plants. Plants 11, 1006. 35448734 10.3390/plants11081006 PMC9029974

[erag105-B11] El Mazlouzi M, Morel C, Robert T, Yan B, Mollier A. 2020. Phosphorus uptake and partitioning in two durum wheat cultivars with contrasting biomass allocation as affected by different P supply during grain filling. Plant and Soil 449, 179–192.

[erag105-B12] Estiarte M, Campioli M, Mayol M, Penuelas J. 2023. Variability and limits of nitrogen and phosphorus resorption during foliar senescence. Plant Communications 4, 100503. 36514281 10.1016/j.xplc.2022.100503 PMC10030369

[erag105-B13] Fang X, Yang D, Deng L, et al 2024. Phosphorus uptake, transport, and signaling in woody and model plants. Forestry Research 4, e017. 39524430 10.48130/forres-0024-0014 PMC11524236

[erag105-B14] FAO . 2020. FAOSTAT. Rome: food and agriculture organization of the united nations. http://www.fao.org/faostat/en

[erag105-B15] Fixen PE . 2009. World fertilizer nutrient reserves - a view to the future. Better Crops with Plant Food 93, 8–11. 10.1002/jsfa.4532 22415449

[erag105-B16] Gao W, Lu L, Wenmin Q, Wang C, Shou H. 2017. *OsPAP26* encodes a major purple acid phosphatase and regulates phosphate remobilization in rice. Plant & Cell Physiology 58, 885–892. 28371895 10.1093/pcp/pcx041

[erag105-B17] Gerdol R, Iacumin P, Brancaleoni L. 2019. Differential effects of soil chemistry on the foliar resorption of nitrogen and phosphorus across altitudinal gradients. Functional Ecology 33, 1351–1361.

[erag105-B18] Girondé A, Etienne P, Trouverie J, et al 2015. The contrasting N management of two oilseed rape genotypes reveals the mechanisms of proteolysis associated with leaf N remobilization and the respective contributions of leaves and stems to N storage and remobilization during seed filling. BMC Plant Biology 15, 59. 25848818 10.1186/s12870-015-0437-1 PMC4384392

[erag105-B19] Gregersen PL, Holm PB, Krupinska K. 2008. Leaf senescence and nutrient remobilisation in barley and wheat. Plant Biology 10, 37–49. 18721310 10.1111/j.1438-8677.2008.00114.x

[erag105-B20] Gurumurthy S, Singh J, Basu PS, Meena SK, Rane J, Singh NP, Hazra KK. 2022. Increased significance of chickpea (*Cicer arietinum* L.) senescence trait under water-deficit environment. Environmental Challenges 8, 100565.

[erag105-B21] Hayes P, Turner BL, Lambers H, Laliberté E. 2014. Foliar nutrient concentrations and resorption efficiency in plants of contrasting nutrient-acquisition strategies along a 2-million-year dune chronosequence. The Journal of Ecology 102, 396–410.

[erag105-B22] Horvath SE, Daum G. 2013. Lipids of mitochondria. Progress in Lipid Research 52, 590–614. 24007978 10.1016/j.plipres.2013.07.002

[erag105-B23] Jeong K, Baten A, Waters DLE, Pantoja O, Julia CC, Wissuwa M, Heuer S, Kretzschmar T, Rose TJ. 2017. Phosphorus remobilization from rice flag leaves during grain filling: an RNA-seq study. Plant Biotechnology Journal 15, 15–26. 27228336 10.1111/pbi.12586 PMC5253468

[erag105-B24] Julia C, Wissuwa M, Kretzschmar T, Jeong K, Rose T. 2016. Phosphorus uptake, partitioning and redistribution during grain filling in rice. Annals of Botany 118, 1151–1162. 27590335 10.1093/aob/mcw164 PMC5091725

[erag105-B25] Killingbeck KT . 1996. Nutrients in senesced leaves: keys to the search for potential resorption and resorption proficiency. Ecology 77, 1716–1727.

[erag105-B26] Kobe RK, Lepczyk CA, Iyer M. 2005. Resorption efficiency decreases with increasing green leaf nutrients in a global data set. Ecology 86, 2780–2792.

[erag105-B27] Kou L, Wang H, Gao W, Chen W, Yang H, Li S. 2017. Nitrogen addition regulates tradeoff between root capture and foliar resorption of nitrogen and phosphorus in a subtropical pine plantation. Trees 31, 77–91.

[erag105-B28] Lin G, Gao M, Zeng D-H, Fang Y. 2020. Aboveground conservation acts in synergy with belowground uptake to alleviate phosphorus deficiency caused by nitrogen addition in a larch plantation. Forest Ecology and Management 473, 118309.

[erag105-B29] Luschin-Ebengreuth N, Zechmann B. 2016. Compartment-specific investigations of antioxidants and hydrogen peroxide in leaves of *Arabidopsis thaliana* during dark-induced senescence. Acta Physiologiae Plantarum 38, 133. 27217598 10.1007/s11738-016-2150-6 PMC4859865

[erag105-B30] Meena RS, Das A, Yadav GS, Lal R. 2018. Legumes for soil health and sustainable management. Singapore: Springer.

[erag105-B31] Motomizu S, Wakimoto T, Tôei K. 1983. Spectrophotometric determination of phosphate in river waters with molybdate and malachite green. The Analyst 108, 361–367. 10.1016/0039-9140(84)80269-6 18963579

[erag105-B32] Nordt B, Hensen I, Bucher SF, et al 2021. The PhenObs initiative: a standardised protocol for monitoring phenological responses to climate change using herbaceous plant species in botanical gardens. Functional Ecology 35, 821–834.

[erag105-B33] Pang J, Bansal R, Zhao H, Bohuon E, Lambers H, Ryan MH, Ranathunge K, Siddique KHM. 2018b. The carboxylate-releasing phosphorus-mobilizing strategy can be proxied by foliar manganese concentration in a large set of chickpea germplasm under low phosphorus supply. New Phytologist 219, 518–529. 29756639 10.1111/nph.15200

[erag105-B34] Pang J, Kim H, Boitt G, Ryan M, Wen Z, Lambers H, Sharma M, Mickan B, Gadot G, Siddique KHM. 2022. Root diameter decreases and rhizosheath carboxylates and acid phosphatases increase in chickpea during plant development. Plant and Soil 476, 219–238.

[erag105-B35] Pang J, Zhao H, Bansal R, Bohuon E, Lambers H, Ryan MH, Siddique KHM. 2018a. Leaf transpiration plays a role in phosphorus acquisition among a large set of chickpea genotypes. Plant, Cell & Environment 41, 2069–2079. 10.1111/pce.13139 29315636

[erag105-B36] Paz-Ares J, Puga MI, Rojas-Triana M, Martinez-Hevia I, Diaz S, Poza-Carrión C, Miñambres M, Leyva A. 2022. Plant adaptation to low phosphorus availability: core signaling, crosstalks, and applied implications. Molecular Plant 15, 104–124. 34954444 10.1016/j.molp.2021.12.005

[erag105-B37] Raven JA, Lambers H, Smith SE, Westoby M. 2018. Costs of acquiring phosphorus by vascular land plants: patterns and implications for plant coexistence. New Phytologist 217, 1420–1427. 29292829 10.1111/nph.14967

[erag105-B38] R Core Team . 2023. R: a language and environment for statistical computing. Vienna, Austria: R Foundation for Statistical Computing.

[erag105-B39] Reichert T, Rammig A, Fuchslueger L, Lugli LF, Quesada CA, Fleischer K. 2022. Plant phosphorus-use and -acquisition strategies in Amazonia. New Phytologist 234, 1126–1143. 35060130 10.1111/nph.17985

[erag105-B40] Sharma SB, Sayyed RZ, Trivedi MH, Gobi TA. 2013. Phosphate solubilizing microbes: sustainable approach for managing phosphorus deficiency in agricultural soils. SpringerPlus 2, 587. 25674415 10.1186/2193-1801-2-587 PMC4320215

[erag105-B41] Shi L, De Biolley L, Shaikh MA, De Vries ME, Mittmann SU, Visser RGF, Prat S, Bachem CWB. 2024. Aging later but faster: how *StCDF1* regulates senescence in *Solanum tuberosum*. New Phytologist 242, 2541–2554. 38197194 10.1111/nph.19525

[erag105-B42] Smith AP, Fontenot EB, Zahraeifard S, DiTusa SF. 2015. Molecular components that drive phosphorus-remobilisation during leaf senescence. In: Roberts JA, ed. Annual plant reviews online. Chichester, UK: John Wiley & Sons, Ltd, 159–186.

[erag105-B43] Song H, Yin Z, Chao M, Ning L, Zhang D, Yu D. 2014. Functional properties and expression quantitative trait loci for phosphate transporter *GmPT1* in soybean. Plant, Cell & Environment 37, 462–472. 10.1111/pce.12170 23889314

[erag105-B44] Srinivasarao C, Ganeshamurthy AN, Ali M, Venkateswarlu B. 2006. Phosphorus and micronutrient nutrition of chickpea genotypes in a multi-nutrient-deficient typic ustochrept. Journal of Plant Nutrition 29, 747–763.

[erag105-B45] Stekhoven DJ, Bühlmann P. 2012. MissForest—non-parametric missing value imputation for mixed-type data. Bioinformatics 28, 112–118. 22039212 10.1093/bioinformatics/btr597

[erag105-B46] Stigter KA, Plaxton WC. 2015. Molecular mechanisms of phosphorus metabolism and transport during leaf senescence. Plants 4, 773–798. 27135351 10.3390/plants4040773 PMC4844268

[erag105-B47] Suárez N . 2010. Leaf lifetime photosynthetic rate and leaf demography in whole plants of *Ipomoea pes-caprae* growing with a low supply of calcium, a ‘non-mobile’ nutrient. Journal of Experimental Botany 61, 843–855. 20080828 10.1093/jxb/erp351 PMC2814114

[erag105-B48] Suriyagoda LDB, Ryan MH, Gille CE, Dayrell RLC, Finnegan PM, Ranathunge K, Nicol D, Lambers H. 2023. Phosphorus fractions in leaves. New Phytologist 237, 1122–1135. 36328763 10.1111/nph.18588

[erag105-B49] Thudi M, Chen Y, Pang J, et al 2021. Novel genes and genetic loci associated with root morphological traits, phosphorus-acquisition efficiency and phosphorus-use efficiency in chickpea. Frontiers in Plant Science 12, 636973. 34122467 10.3389/fpls.2021.636973 PMC8192852

[erag105-B50] Treseder KK, Vitousek PM. 2001. Effects of soil nutrient availability on investment in acquisition of N and P in Hawaiian rain forests. Ecology 82, 946–954.

[erag105-B51] Tsujii Y, Onoda Y, Kitayama K. 2017. Phosphorus and nitrogen resorption from different chemical fractions in senescing leaves of tropical tree species on Mount Kinabalu, Borneo. Oecologia 185, 171–180. 28871400 10.1007/s00442-017-3938-9

[erag105-B52] Tully KL, Wood TE, Schwantes AM, Lawrence D. 2013. Soil nutrient availability and reproductive effort drive patterns in nutrient resorption in *Pentaclethra macroloba*. Ecology 94, 930–940.

[erag105-B53] Varshney RK, Roorkiwal M, Sun S, et al 2021. A chickpea genetic variation map based on the sequencing of 3,366 genomes. Nature 599, 622–627. 34759320 10.1038/s41586-021-04066-1 PMC8612933

[erag105-B54] Veneklaas EJ . 2022. Phosphorus resorption and tissue longevity of roots and leaves – importance for phosphorus use efficiency and ecosystem phosphorus cycles. Plant and Soil 476, 627–637.

[erag105-B55] Veneklaas EJ, Lambers H, Bragg J, et al 2012. Opportunities for improving phosphorus-use efficiency in crop plants. New Phytologist 195, 306–320. 22691045 10.1111/j.1469-8137.2012.04190.x

[erag105-B56] Vergutz L, Manzoni S, Porporato A, Novais RF, Jackson RB. 2012. Global resorption efficiencies and concentrations of carbon and nutrients in leaves of terrestrial plants. Ecological Monographs 82, 205–220.

[erag105-B57] Wang D, Freschet GT, McCormack ML, Lambers H, Gu J. 2025. Nutrient resorption of leaves and roots coordinates with root nutrient-acquisition strategies in a temperate forest. New Phytologist 246, 515–527. 39931837 10.1111/nph.70001

[erag105-B58] Wang F, Rose T, Jeong K, Kretzschmar T, Wissuwa M. 2016. The knowns and unknowns of phosphorus loading into grains, and implications for phosphorus efficiency in cropping systems. Journal of Experimental Botany 67, 1221–1229. 26662950 10.1093/jxb/erv517

[erag105-B59] Wen Z, Pang J, Tueux G, Liu Y, Shen J, Ryan MH, Lambers H, Siddique KHM. 2020. Contrasting patterns in biomass allocation, root morphology and mycorrhizal symbiosis for phosphorus acquisition among 20 chickpea genotypes with different amounts of rhizosheath carboxylates. Functional Ecology 34, 1311–1324.

[erag105-B60] Wen Z, Pang J, Wang X, et al 2023. Differences in foliar phosphorus fractions, rather than in cell-specific phosphorus allocation, underlie contrasting photosynthetic phosphorus use efficiency among chickpea genotypes. Journal of Experimental Botany 74, 1974–1989. 36575916 10.1093/jxb/erac519

[erag105-B61] Wetzels M, Odekerken-Schröder G, Van Oppen C. 2009. Using PLS path modeling for assessing hierarchical construct models: guidelines and empirical illustration. MIS Quarterly 33, 177.

[erag105-B62] Wright IJ, Westoby M. 2003. Nutrient concentration, resorption and lifespan: leaf traits of Australian sclerophyll species. Functional Ecology 17, 10–19.

[erag105-B63] Wu X-Y, Hu W-J, Luo H, Xia Y, Zhao Y, Wang L-D, Zhang L-M, Luo J-C, Jing H-C. 2016. Transcriptome profiling of developmental leaf senescence in sorghum (*Sorghum bicolor*). Plant Molecular Biology 92, 555–580. 27586543 10.1007/s11103-016-0532-1

[erag105-B64] Yang G, Deng M, Guo L, Du E, Zheng Z, Peng Y, Zhao C, Liu L, Yang Y. 2025. Characteristics of leaf nutrient resorption efficiency in Tibetan alpine permafrost ecosystems. Nature Communications 16, 4044. 10.1038/s41467-025-59289-x PMC12041207 40301364

[erag105-B65] Yang M, Lu J, Liu M, Lu Y, Yang H. 2020. Leaf nutrient resorption in lucerne decreases with relief of relative soil nutrient limitation under phosphorus and potassium fertilization with irrigation. Scientific Reports 10, 10525. 32601320 10.1038/s41598-020-65484-1 PMC7324584

[erag105-B66] Yang S-Y, Lin W-Y, Hsiao Y-M, Chiou T-J. 2024. Milestones in understanding transport, sensing, and signaling of the plant nutrient phosphorus. The Plant Cell 36, 1504–1523. 38163641 10.1093/plcell/koad326 PMC11062440

[erag105-B67] Yu L, Huang Z, Li Z, Korpelainen H, Li C. 2022. Sex-specific strategies of nutrient resorption associated with leaf economics in *Populus euphratica*. The Journal of Ecology 110, 2062–2073.

[erag105-B68] Zhao Q, Guo J, Shu M, Wang P, Hu S. 2020. Impacts of drought and nitrogen enrichment on leaf nutrient resorption and root nutrient allocation in four Tibetan plant species. The Science of the Total Environment 723, 138106. 32222509 10.1016/j.scitotenv.2020.138106

