# Supplementary material for: Carboxylate exudation and reproductive effort are associated with leaf phosphorus-resorption efficiency in chickpea
Source: J Exp Bot. 2026 Feb 27;77(12):3970–83. doi: 10.1093/jxb/erag105 (PMC13293075; doi:10.1093/jxb/erag105)
Supplement: erag105_Supplementary_Data [file erag105_supplementary_data.pdf]

Supplementary material

Carboxylate exudation and reproductive efforts are associated with leaf phosphorus-resorption efficiency in chickpea

Xiaolong Feng[1,3*], Huaikang Jing[1,3], Chuangwei Fang[1,3], Gabriel Crepin[1,2], Augustin Dusannier[1,2], Jiayin Pang[1,3*], Peta L. Clode[1,4], Kadambot H. M. Siddique[3], Hans Lambers[1,3]

* Correspondence:

xiaolong.feng@research.uwa.edu.au,

jiayin.pang@uwa.edu.au

Supporting figures and tables (Tables S1; Figures S1-S12):

Table S1 Composition of basal nutrients applied before planting. Soil nutrient concentrations are indicated for each added nutrient.

**Added nutrients**	**Nutrient**	**Soil nutrient concentration (mg kg^{-1} soil)**
$Ca(NO_3)_2 \cdot 4H_2O$	Ca	
	N	11.25
NH_4Cl	N	3.75
K_2SO_4	K	109.6875
	S	45
$ZnSO_4 \cdot 7H_2O$	Zn	2
	S	1
$CuSO_4 \cdot 5H_2O$	Cu	0.5
	S	0.25
H_3BO_3	B	0.12
$NaMoO_4 \cdot 2H_2O$	Mo	0.40
FeNaEDTA	Fe	3
KH_2PO_4	P	20
	K	22.65

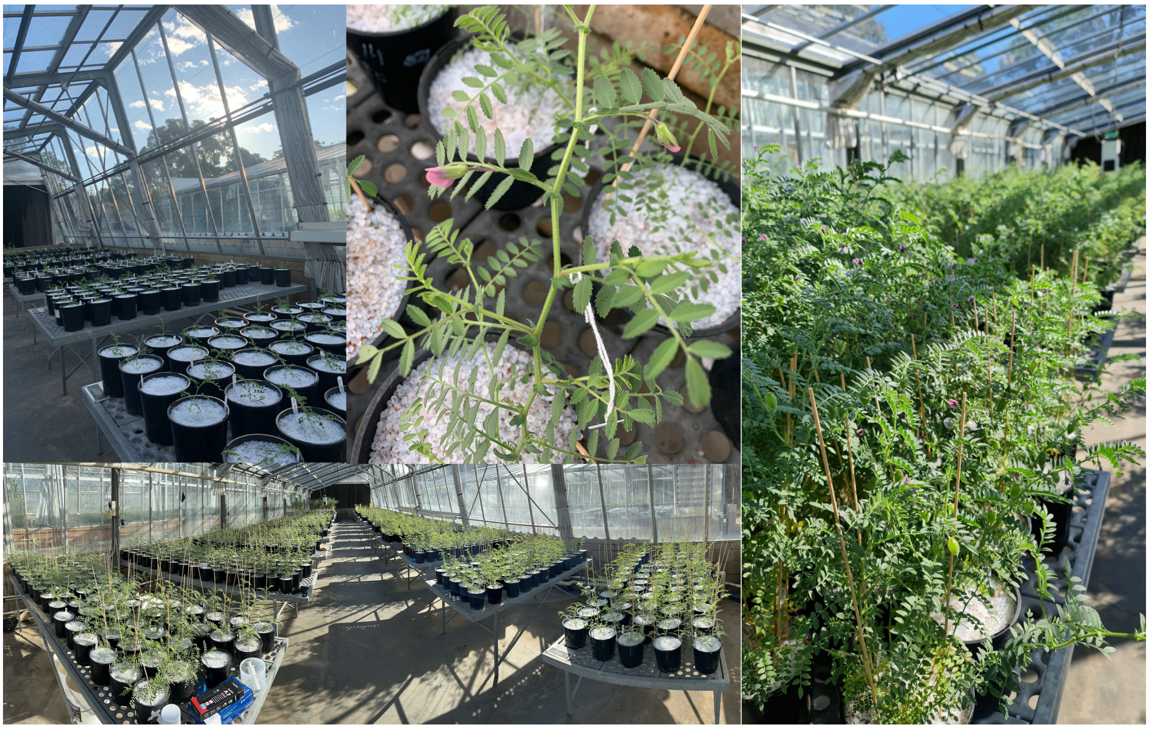

Fig. S1 Cultivation of experimental plants in controlled greenhouse conditions.

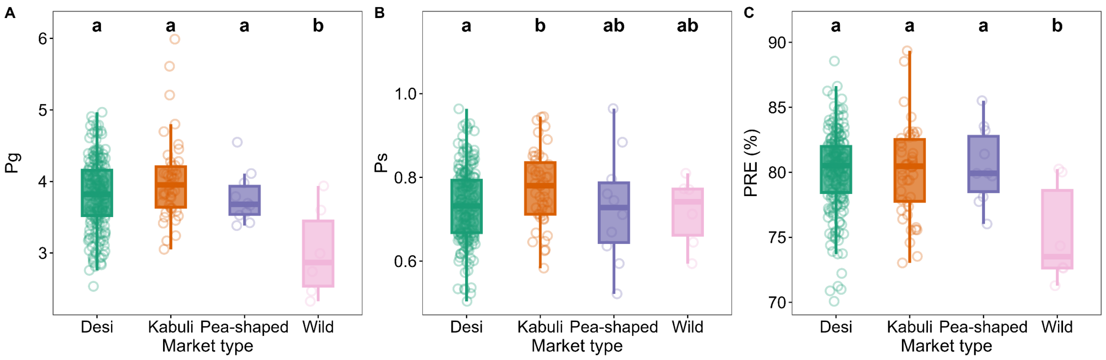

Fig. S2 Box plots showing (A) green leaf phosphorus (P) concentration (P_g), (B) senesced leaf P concentration (P_s), and (C) P-resorption efficiency (PRE) across 266 chickpea accessions including 203 desi types, 47 kabuli types, 10 pea-shaped types and six wild accessions (two *Cicer* species) supplied with 20 µg P g^{-1} soil in a glasshouse. Different lowercase letters indicate significant differences among market types (Tukey's HSD test, P < 0.05).

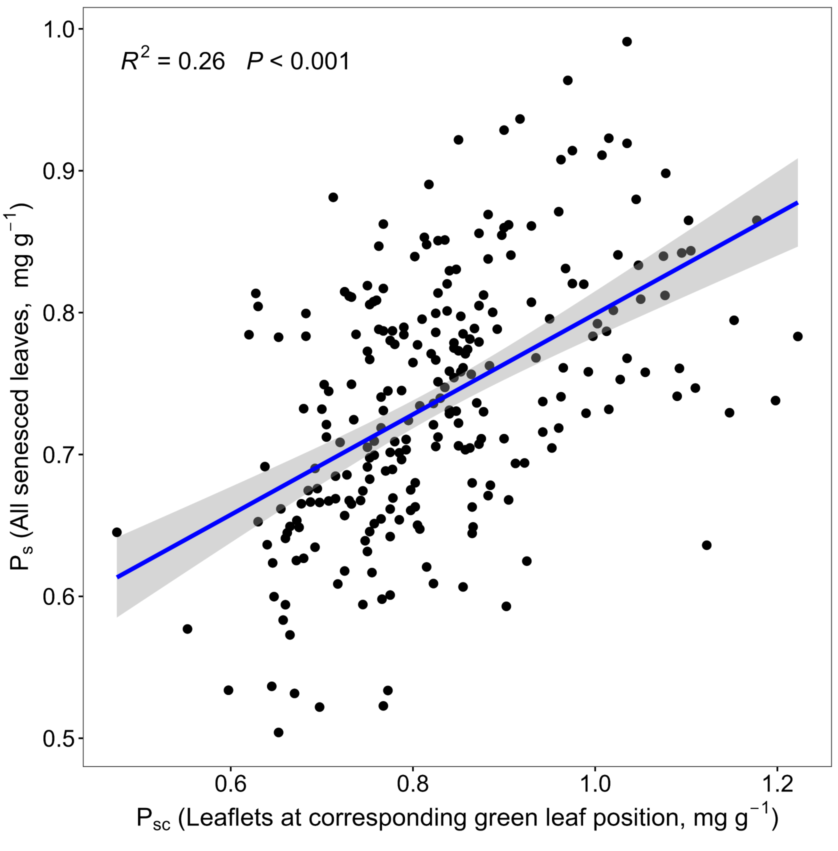

Fig. S3 Correlation between the phosphorus (P) concentration of all senesced leaves (P_s) and senesced leaflets at the corresponding green leaf position (P_{sc}) across 260 cultivated chickpea accessions supplied with 20 µg P g^{-1} soil in a glasshouse experiment. The solid line represents a linear regression fit, and the shaded area indicates the 95% confidence interval.

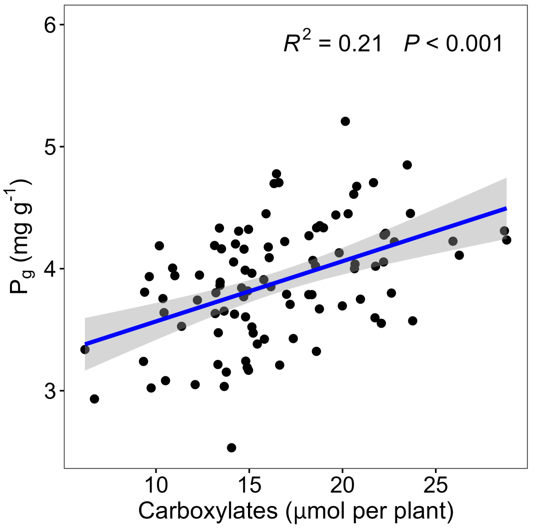

Fig. S4 Correlation between green leaf phosphorus (P) concentration (Pg) and rhizosheath carboxylate release per plant across 98 chickpea accessions (data from Pang et al. (2018b)). The solid line represents a linear regression fit; shaded area indicates 95% confidence interval.

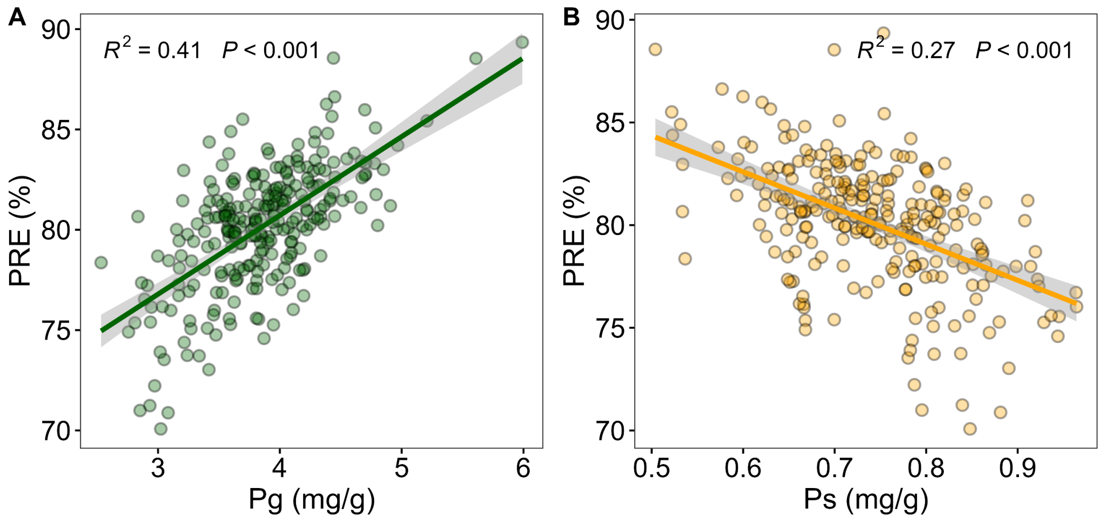

Fig. S5 Correlations between phosphorus (P)-resorption efficiency (PRE) and P concentration in green (P_g) and senesced (P_s) leaves across 260 cultivated chickpea accessions supplied with 20 μg P g^{-1} soil in a glasshouse. Solid lines represent linear regression fits; shaded areas indicate 95% confidence intervals.

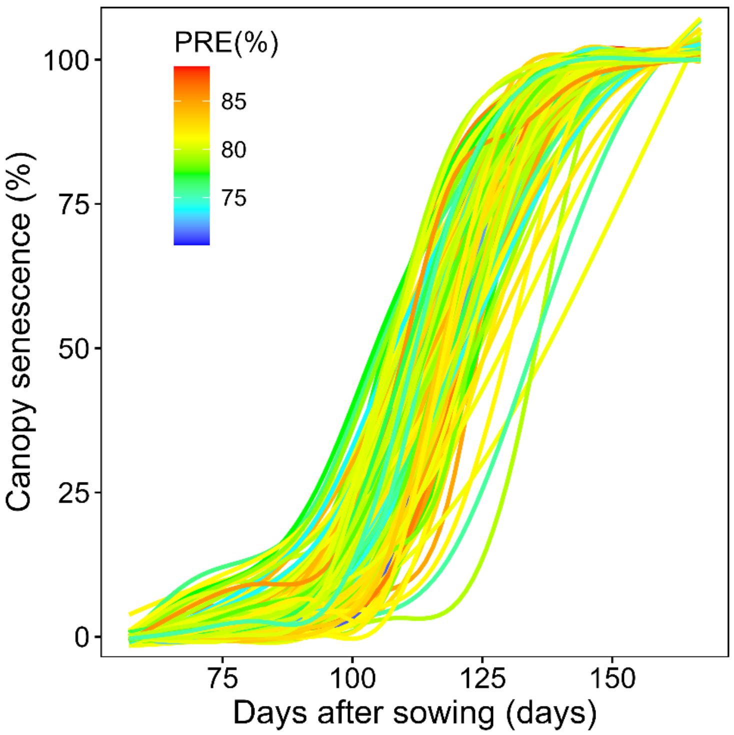

Fig. S6 Changes in the percentage of canopy senescence during plant development across 260 cultivated chickpea accessions supplied with 20 µg P g^{-1} soil in a glasshouse. Different colours indicate different accessions with varying phosphorus-resorption efficiency (PRE). Canopy senescence dynamics were fitted using a generalised additive model (GAM), with the time corresponding to 50% canopy senescence defined as T_{c50}.

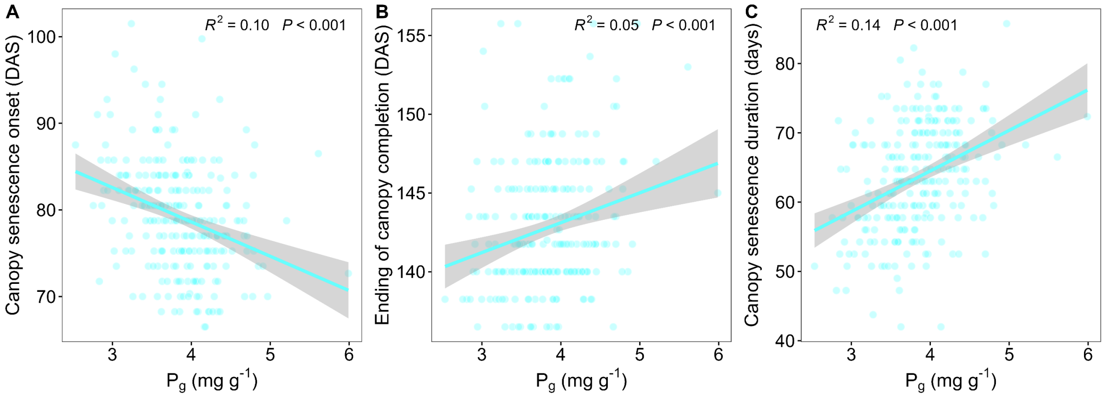

Fig. S7 Canopy senescence (A) onset, (B) completion and (C) duration in relation to green leaf phosphorus (P) concentration (P_g) across 260 cultivated chickpea accessions supplied with 20 µg P g^{-1} soil in a glasshouse. DAS = days after sowing. Solid lines indicate linear regression fits; shaded areas represent 95% confidence intervals.

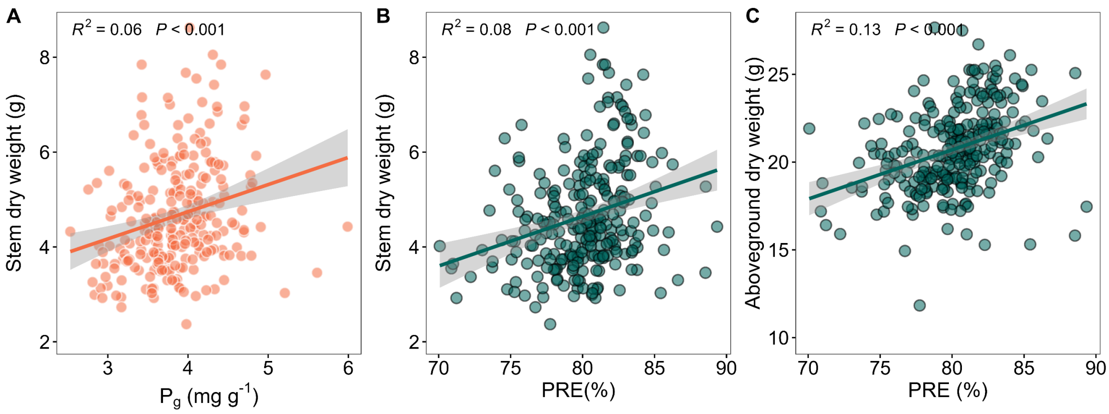

Fig. S8 Correlations between green leaf phosphorus (P) concentration (P_g) and (A) stem dry weight and between phosphorus-resorption efficiency (PRE) and (B) total stem and (C) aboveground dry weight across 260 cultivated chickpea accessions supplied with 20 μg P g^{-1} soil in a glasshouse. Solid lines indicate linear regression fits; shaded areas represent 95% confidence intervals.

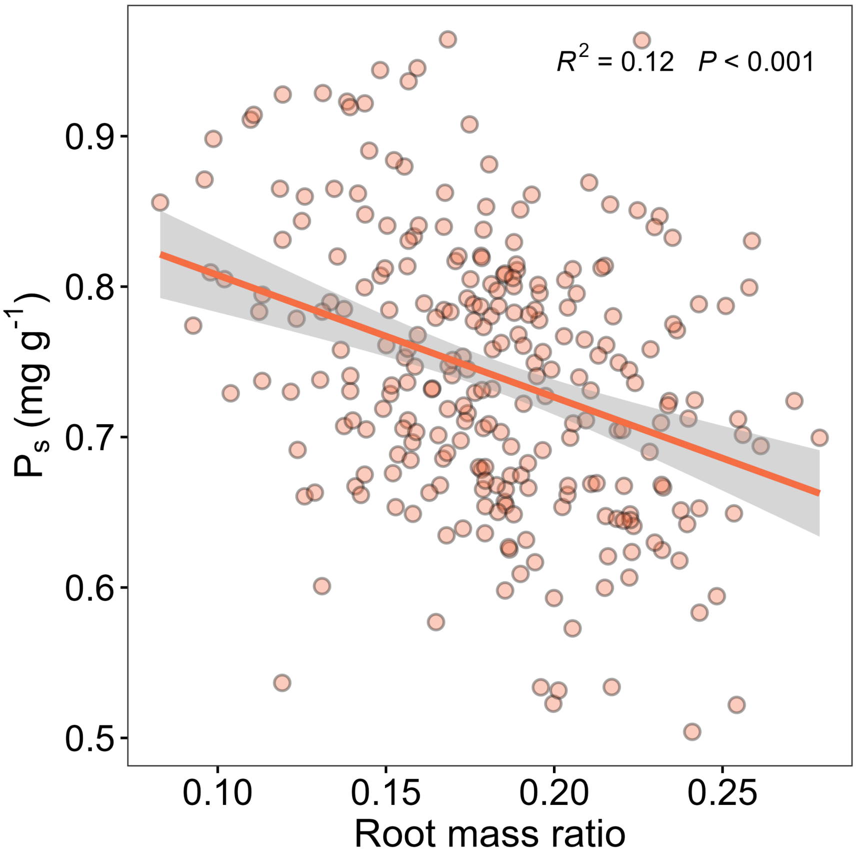

Fig. S9 Relationship between senesced leaf phosphorus (P) concentration (P_s) and root mass ratio across 260 cultivated chickpea accessions supplied with 20 μg P g^{-1} soil in a glasshouse. Solid lines indicate linear regression fits; shaded areas represent 95% confidence intervals.

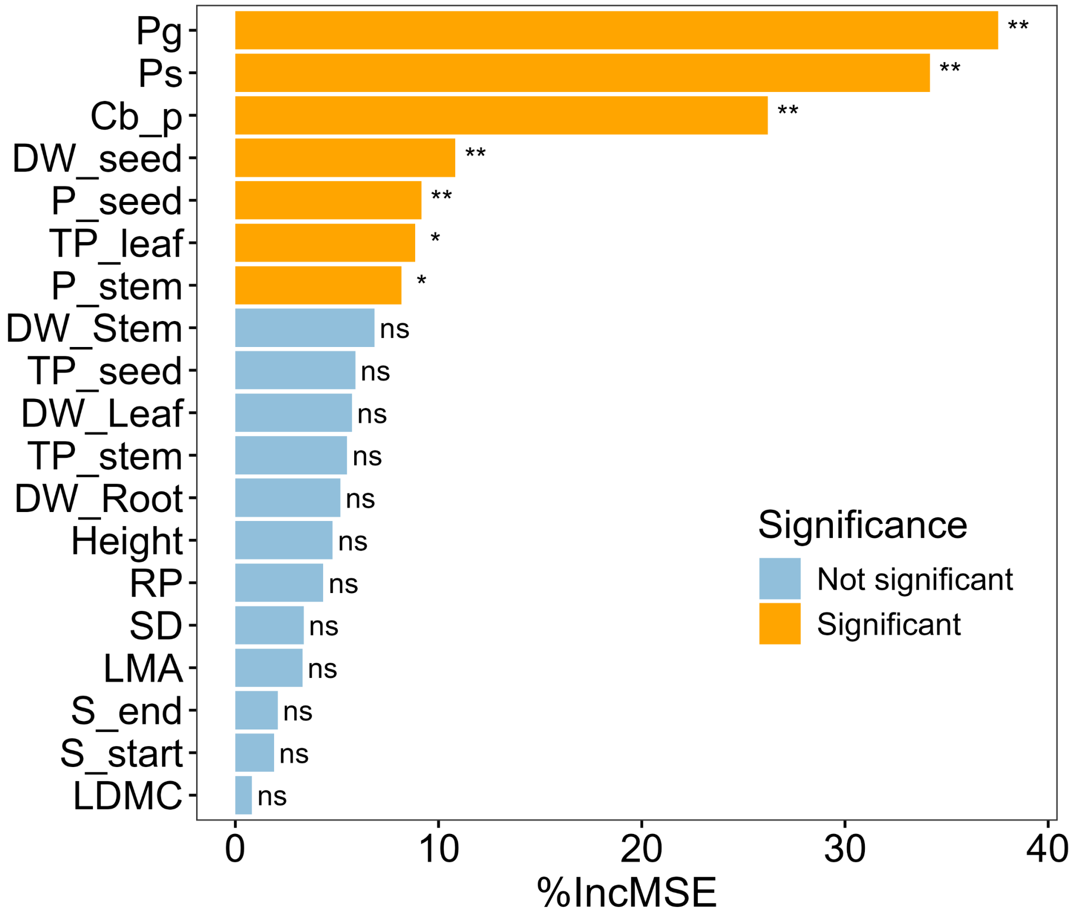

Fig. S10 Results of the random forest model predicting phosphorus-resorption efficiency across 260 cultivated chickpea accessions supplied with 20 μg P g^{-1} soil in a glasshouse.

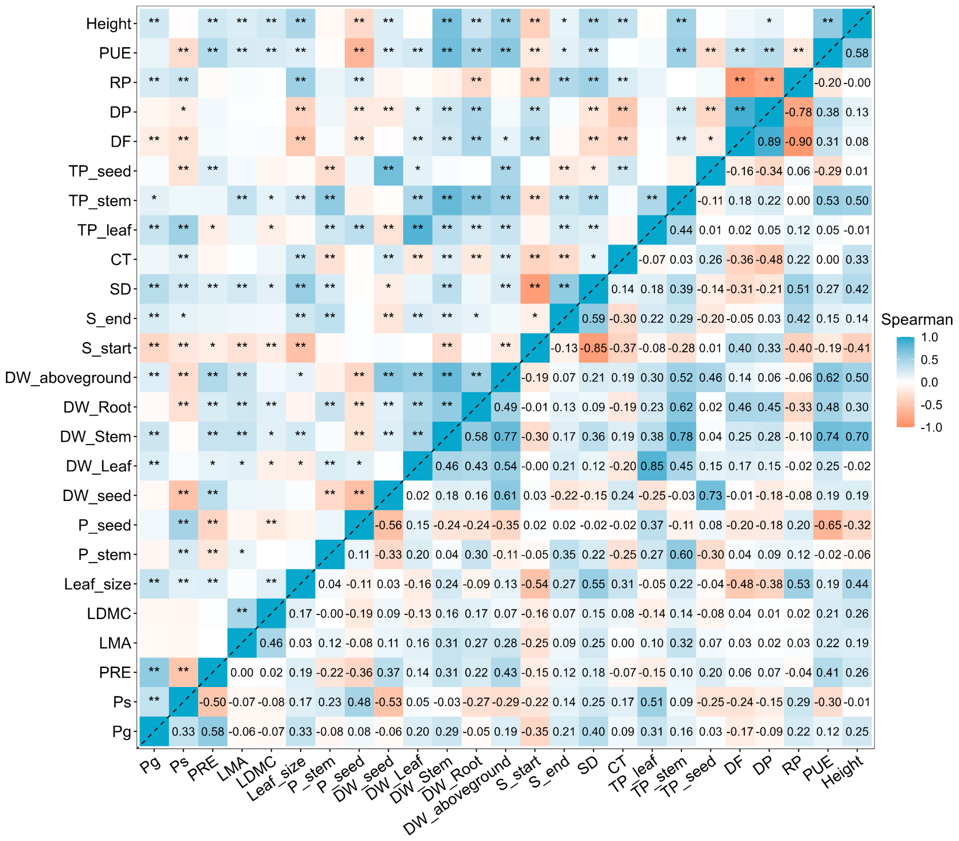

Fig. S11 Pairwise Spearman correlation among 25 traits measured across 260 chickpea accessions supplied with 20 µg phosphorus (P) g^{-1} soil in a glasshouse experiment.

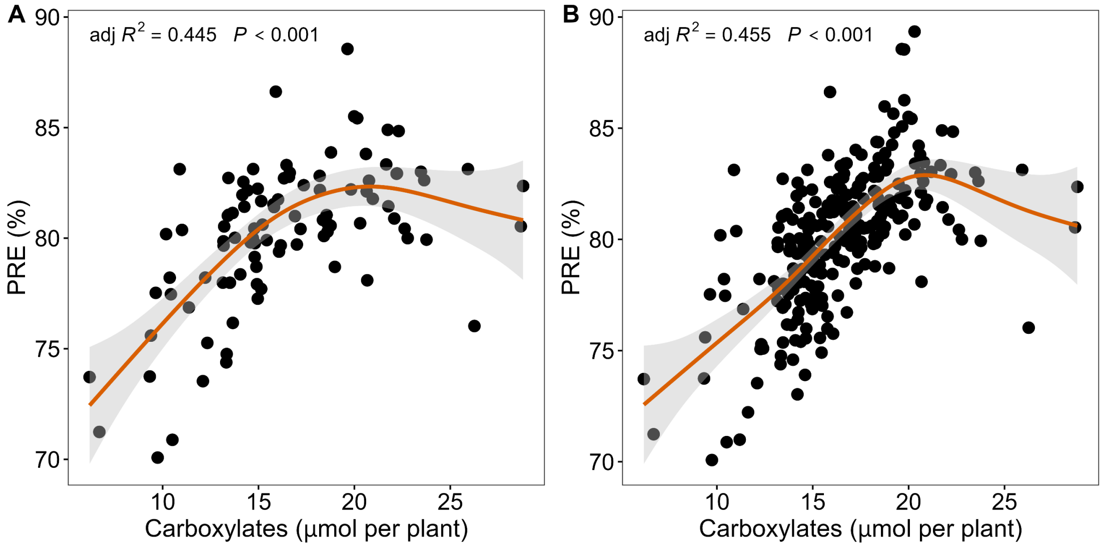

Fig. S12 Correlation between phosphorus-resorption efficiency (PRE) and rhizosheath carboxylate release per plant across 260 chickpea accessions under original (A) and simulated (B) conditions. The correlation was modelled using a generalised additive model (GAM). The solid line represents the GAM fit; the shaded area indicates 95% confidence intervals.
